# Landscape, Evidence, Gaps, and Opportunities in Digital Mental Health Interventions for Older Adults: Scoping Review

**DOI:** 10.2196/92542

**Published:** 2026-07-06

**Authors:** Leyi Zhou, Xinyi Zuo, Joonyoung Cho, Chuxuan Zheng, Shengyi Jing, Xiaoling Xiang

**Affiliations:** 1 School of Social Welfare, University of California, Berkeley, Berkeley, CA, United States; 2 School of Social Work, University of Michigan, 1080 South University AvenueAnn Arbor, MI, 48109, United States, 1 734 763 6581; 3 Center on Aging, Thompson School of Social Work and Public Health, University of Hawaii System, Honolulu, HI, United States; 4 Department of Psychology, College of Literature, Science, and the Arts, University of Michigan, Ann Arbor, MI, United States

**Keywords:** digital mental health interventions, gerontechnology, scoping review, age-adaptation design, cognitive behavioral therapy

## Abstract

**Background:**

Mental health conditions, including depression, anxiety, and psychological distress, are prevalent among the aging population and affect their health, functioning, and quality of life. Access to proper and high-quality mental health treatment is necessary; however, mental health treatment and care remain underused due to stigma, workforce shortages, cost, and mobility limitations. Digital mental health interventions (DMHIs) are emerging as a promising strategy to improve the accessibility and effectiveness of mental health services for older adults, but older adults have historically been underrepresented in DMHI development and evaluation. Additionally, the effectiveness of different types of DMHIs and how age-centered design approaches influence outcomes remain underexplored.

**Objective:**

This scoping review mapped and synthesized evidence on DMHIs focused on adults aged 50 years and older and identified gaps in the evidence base related to study design, age-related adaptations, and clinical outcomes. Specifically, we examined (1) the technologies and therapeutic approaches used, (2) the outcomes and effectiveness of DMHIs, and (3) age-centered adaptations and their outcomes.

**Methods:**

This scoping review searched for studies focusing on DMHIs for older adults across PubMed, PsycINFO, Scopus, Ageline, and Web of Science that were published from 2000 to February 2025. Eligible studies evaluated or described the design of DMHIs targeting mental health conditions among adults aged 50 years or older. Two rounds of independent screening and data extraction were conducted by multiple reviewers. Extracted data included study design, sample characteristics, intervention features, technologies used, age-related adaptations, and clinical outcomes.

**Results:**

Seventy-two studies met the inclusion criteria, of which 36 were randomized controlled trials and 54 reported clinical outcomes. Web-based cognitive behavioral therapy was the most commonly used approach, followed by games, virtual reality, mobile apps, chatbots, and robots. Fifty-four studies reported positive clinical outcomes, most commonly reductions in depression, anxiety, or psychological distress. However, only one-third of the studies incorporated age-centered design adaptations or co-design approaches, such as simplified interfaces, larger fonts, age-relevant content, or participatory development with older adults.

**Conclusions:**

Among studies reporting positive clinical outcomes, DMHIs can reduce depression, anxiety, and psychological distress. However, with only half of the included studies using randomized controlled trial designs, the overall evidence base remains moderate. In addition, age-adaptive design remains underdeveloped. Future research should strengthen trial designs and systematically examine how usability and age-centered adaptations influence DMHI effectiveness.

## Introduction

Mental health conditions, such as depression and anxiety, are prevalent among older adults and can significantly impact their health and quality of life [1]. Although many evidence-based mental health treatments exist, they are persistently underused among older adults [1]. Factors such as stigma [2], transportation barriers [3], a shortage of mental health professionals [4], financial constraints [5], and insufficient insurance coverage [6] are common access barriers faced by many vulnerable populations needing mental health treatments. These access barriers are compounded in older adults due to misconceptions about mental health conditions, such as the belief that depression is a normal part of aging [7], challenges in identifying and diagnosing certain medical health conditions in later life [1], and more pervasive mental health stigma [8], as well as reluctance to seek help [9].

Digital mental health interventions (DMHIs) are a promising solution to address the growing unmet mental health needs among older adults. Various technologies have been used to deliver mental health treatments, such as smartphone apps, telecommunication tools (eg, SMS text messaging, emailing, and video conferencing), online platforms, computer-based programs, remote monitoring devices, wearable technologies, and virtual reality (VR) [10-13]. DMHIs have been hailed for their scalability, accessibility, efficiency, and potential cost-effectiveness [10,14]. Many DMHIs can be used repeatedly with different patients without losing their therapeutic power and at a reduced marginal cost, making them especially helpful in underresourced settings [15]. Many DMHIs are self-guided, which may provide more privacy for those with internalized stigma toward mental illness and make them more receptive to treatment [16]. In addition, many DMHIs can be accessed anywhere with an internet connection, thus eliminating the need for attending office-based treatments, a significant barrier for those with transportation barriers due to mobility difficulties or driving cessation [17]. Some DMHI modalities, such as internet-based cognitive behavioral therapy (CBT), have a robust body of evidence supporting their effectiveness in mixed-age samples and diverse settings [18-20].

Although the potential of DMHIs is increasingly recognized, older adults have traditionally been underrepresented and sometimes excluded from DMHI development and testing. Despite the age-related digital divide, older adults have increasingly used technologies for health promotion and maintenance, particularly since the COVID-19 pandemic [21]. The increased adoption and acceptance of technology have paved the way for a wider uptake of DMHIs among older adults. Indeed, we have observed an uptick in DMHI studies focused on older adults, including those from socioeconomically disadvantaged backgrounds [22-24]. Given the fast pace of technological advancement and continued interest in DMHIs, a comprehensive and up-to-date review is essential to pinpoint existing gaps and suggest future directions.

The purpose of this scoping review was to systematically scope and map existing evidence on DMHIs focused on older adults. Previous review papers have focused on DMHI trials to evaluate whether they are clinically effective in reducing depression, anxiety, or other mental health symptoms among older adults [25,26]. Different from reviews that primarily provide evidence on intervention effectiveness, this review emphasizes that the success of DMHIs for older adults depends not only on clinical efficacy but also on usability, age-relevant content, and fit with older adults’ needs. This review provides more insight into how DMHIs are designed, how age-centered adaptations are incorporated, and how these adaptations may contribute to positive clinical outcomes. Our goal is to move beyond simply asking whether DMHIs benefit older adults. Instead, we aim to stimulate discussions around which program features are most likely to induce behavioral change, identify opportunities for treatment innovation, and provide insights for designing future DMHIs that better meet the needs of older adults.

To this end, this scoping review aimed to (1) describe the types of technologies and program features used in DMHIs for treating and preventing mental health conditions in older adults, (2) assess the range and nature of procedural, content, and technological adaptations made in DMHIs specifically for older adults, and (3) qualitatively explore and summarize the clinical outcomes of DMHIs across various technologies and program features.

## Methods

### Study Design

A scoping review fits our study aim well, as it allows for a comprehensive mapping of existing studies, identification of gaps in the current literature, summarization of research activities, and dissemination of findings to inform future research, policy, and practice [27,28]. We carried out the review process following the framework developed by Arksey and O’Malley [27] and later refined by Levac et al [28].

### Search Strategy

Electronic searches for this study were initially conducted in June 2022 across 5 databases: PubMed, PsycINFO, Scopus, Ageline, and Web of Science. An updated literature search was performed in February 2025 to capture new studies published since the initial search. Multimedia Appendix 1 shows the search terms, which were developed based on the PICO (population, intervention, comparator, and outcome) framework: (1) population: studies involving participants aged 50 years or older; (2) intervention: DMHIs providing treatment and support for mental health conditions; (3) comparator: any comparator or no comparator; (4) outcome: types of technologies used, adaptations made for older adults, and the effects of DMHIs.

Our search was limited to publication years from January 2000 to February 2025, as studies on telepsychiatry began to emerge around 2000 [29]. Only studies reported in English were included. The references of the included studies and relevant review papers were checked for additional relevant studies. If full-text studies were not available, the corresponding authors were contacted. The initial search process was carried out by the first author, and duplicates were removed prior to screening. The inclusion and exclusion criteria, as well as data extraction forms, were drafted by the first author and the corresponding author.

### Inclusion and Exclusion Criteria

Table 1 presents the inclusion and exclusion criteria for study selection. Studies were included if they: (1) were empirical studies (qualitative or quantitative) evaluating the outcomes of DMHIs or describing the design process of DMHIs; (2) focused on older adults, defined as studies in which all participants were aged 50 years or older, or where analyses were stratified to isolate treatment effects for this age group; (3) involved interventions targeting mental health conditions commonly experienced by older adults; (4) focused on DMHIs, including web-based interventions, software applications, artificial intelligence (AI) tools, video game consoles, or wearable technology aimed at addressing mental health conditions; and (5) were written in English.

**Table 1.** Inclusion and exclusion criteria.

Element	Inclusion criteria	Exclusion criteria
Population	At least any kind of mental health conditions (eg, depression, general psychological distress, psychological distress with physical condition, anxiety, posttraumatic stress disorder, and grief) Aged ≥50 years	Aged <50 years
Intervention	DMHI^a^ (eg, web-based interventions, software apps, artificial intelligence, video game consoles, and wearable technology)	N/A^b^
Comparison	Different interventions (eg, delayed-treatment waitlist control group, waitlist control group, standardized institutional rehabilitation, support from voluntary caregiver associations, placebo control exercise, and conventional care)	N/A
Outcomes	Types of technologies being applied, adaptations to older adults, and effects of DMHIs	N/A
Study design	Empirical studies (qualitative or quantitative) (eg, RCT^c^, single-group studies, observational studies, quasi-experimental studies, and mixed methods studies)	Review articles Protocols Perspective pieces
Language	English papers	Non-English papers
Types of mental health problems	Depression General psychological distress Psychological distress with physical condition Anxiety Posttraumatic stress disorder Grief	Loneliness Isolation Sleep disorder
Type of technology	DMHIs (eg, web-based interventions, software apps, artificial intelligence, video game consoles, and wearable technology for any mental health condition)	Technologies for monitoring mental health symptoms Focused on cognitive training or cognitive rehabilitation with the primary goal of improving cognition or managing symptoms of dementia Chronic disease self-management tools with the primary purpose of improving self-management behaviors among persons with chronic physical health conditions Primarily aimed to improve health and physical functioning, even if they reported improved mental health outcomes Telepsychiatry or tele–mental health studies, where services were provided by phone or teleconferencing tools by a human therapist Focused on electronic health or medical records, decision support tools for clinicians, analytic services, services that primarily provided support and education to health professionals, clinical practice management software, and clinical workflow and communication software

a DMHI: digital mental health interventions.

b N/A: not applicable.

c RCT: randomized controlled trial.

Studies were excluded if they met any of the following criteria: (1) the study sample included individuals under the age of 50 years, and the treatment effects for older adults (≥50 y) were not isolated through stratified analysis; (2) they were review articles, study protocols, or perspective or commentary pieces; (3) the focus was solely on technologies used for monitoring mental health symptoms without a therapeutic component; (4) the primary aim was cognitive training or rehabilitation focused on improving cognition or managing symptoms of dementia; (5) the intervention centered on chronic disease self-management tools with the primary goal of improving self-management behaviors or physical health outcomes, even if mental health outcomes were reported as secondary outcomes; (6) the study focused exclusively on telepsychiatry or tele–mental health interventions delivered by a human therapist through phone or video conferencing; or (7) the study focused on tools such as electronic health or medical records, decision support tools for clinicians, analytic services, educational or support services for health professionals, clinical practice management software, or clinical workflow and communication tools not intended for direct mental health interventions.

In the first round of screening conducted in 2022, 2 reviewers (LZ and JC) independently screened titles and abstracts using the inclusion and exclusion criteria outlined above. A second round was carried out by 2 coauthors (LZ and SJ) in 2025, following the same procedure. Studies meeting all inclusion criteria were retained, and those meeting any exclusion criteria were removed. Disagreements between reviewers were resolved after consultation with the corresponding author.

### Data Extraction and Synthesis

Three reviewers (LZ, JC, and CZ) independently extracted data, with discrepancies resolved by the corresponding author. Data were entered and managed in an electronic database. Extracted data included the country of study, recruitment settings, study design, sample characteristics (eg, sample size, mean age, gender composition, and education level), mental health condition of focus, types of technologies, intervention features (eg, mode of delivery, type of device required, theoretical underpinnings, and treatment duration), clinical outcomes, and a narrative summary of key findings.

We summarized study characteristics and created cross-tabulations of key characteristics to conduct a qualitative synthesis and identify common themes. Specifically, we examined the distinctive features of different types of DMHIs, the mental health conditions they targeted, and the extent to which these technologies improved participants’ experiences and outcomes.

## Results

### Study Selection

**Figure 1.**
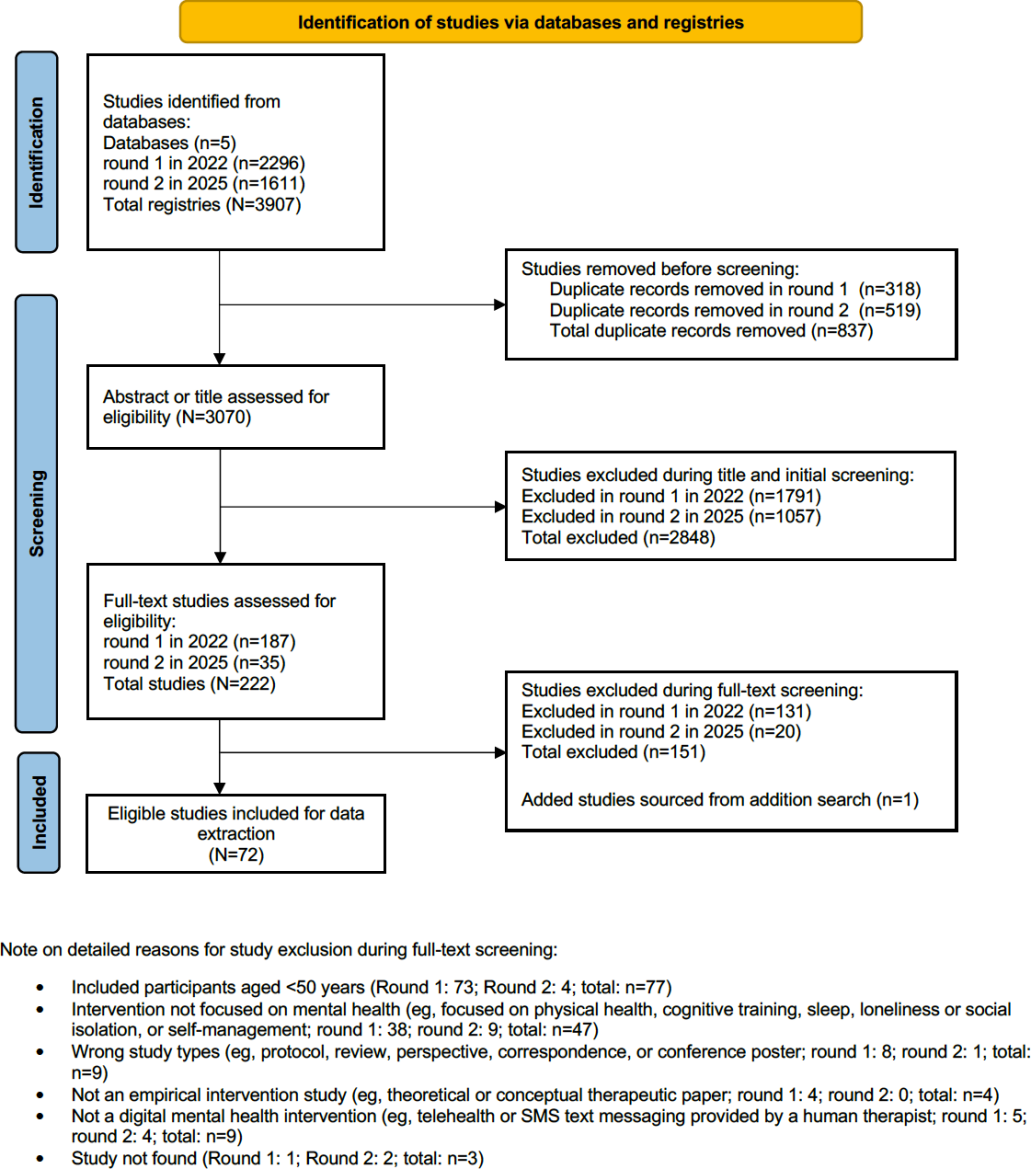
PRISMA (Preferred Reporting Items for Systematic Reviews and Meta-Analyses) flow diagram.

Figure 1 presents the PRISMA (Preferred Reporting Items for Systematic Reviews and Meta-Analyses) flow diagram of the screening process. This scoping review was conducted and reported in accordance with the PRISMA-ScR (Preferred Reporting Items for Systematic Reviews and Meta-Analyses Extension for Scoping Reviews) guidelines (Checklist 1). A total of 3907 potentially relevant studies were identified across rounds 1 and 2, with 3070 remaining after duplicates were removed. Title and abstract screening yielded 222 studies for full-text review. After the full-text review, 72 studies were included in this scoping review. The screening process was managed using the Rayyan platform (Rayyan Systems Inc).

### Main Characteristics of the Included Studies

**Figure 2.**
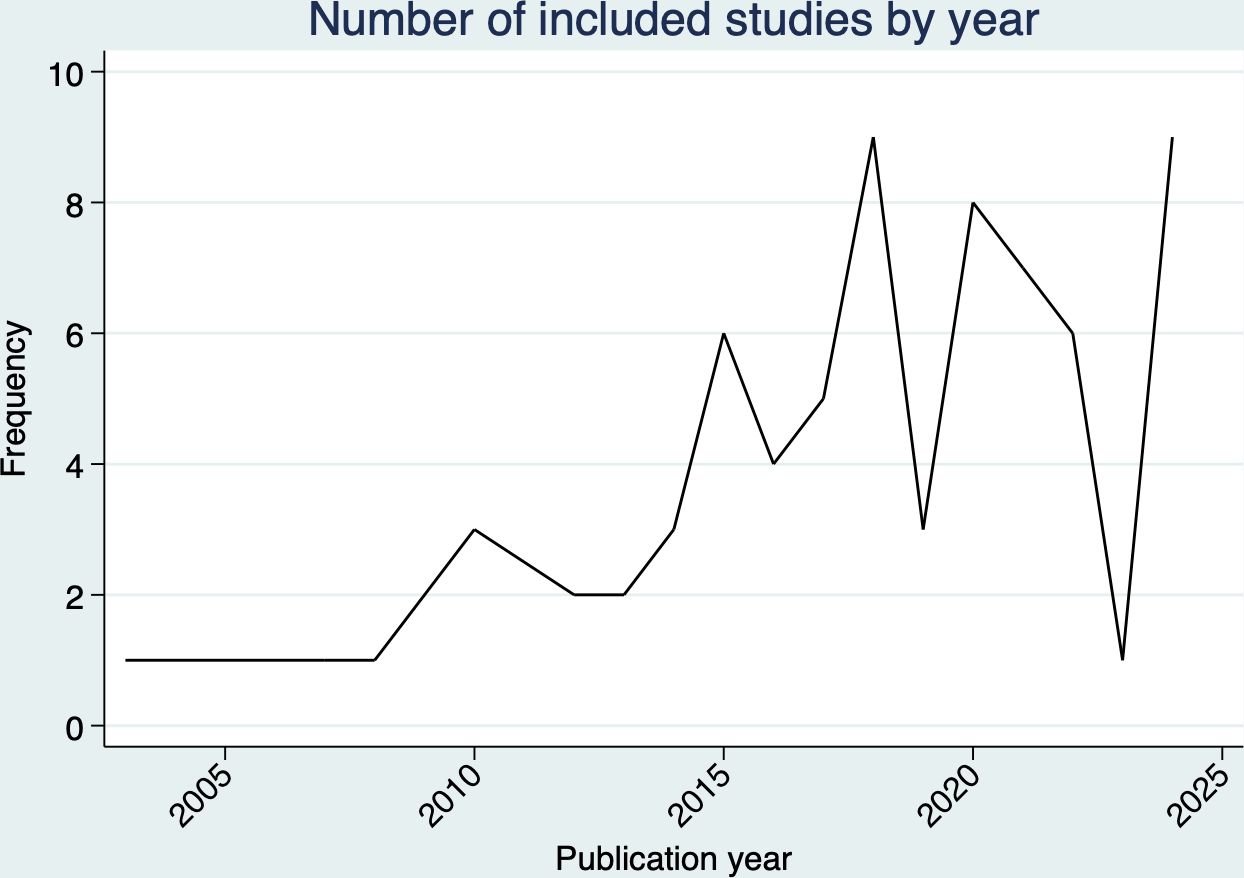
Number of included studies published each year.

Among the 72 included studies, the majority were published after 2015. Table 2 and Figure 2 illustrate an increasing trend in publications since 2020, reflecting the significant expansion and adoption of digital mental health solutions since the COVID-19 pandemic [30].

**Table 2.** Number of included studies per year (N=72).

Year group	Value, n (%)
Before and in 2004	1 (1.39)
2005‐2009	3 (4.17)
2010‐2014	10 (13.89)
2015‐2019	27 (37.50)
2020‐2025	31 (43.06)

More detailed study characteristics are presented in Multimedia Appendix 2. The intervention took place across 18 countries, with the largest number of studies conducted in the United States (n=31), followed by Australia (n=10). Other top countries included China, Sweden, Italy, and Finland. This indicates that while the United States and Australia have a strong presence, interest in DMHIs is growing globally. That said, most of these countries represent the Global North. The sample sizes of the included studies ranged from as few as 3 participants to over 900. Most studies targeted older adults aged 60 years and above, with mean sample ages ranging from 55 to 88.2 years. Of the 72 studies that reported gender composition, 65 included more than 50% female participants. Most studies recruited participants through a variety of channels, including long-term care agencies, clinical settings, community centers, and online platforms or websites. Completion rates were generally high, with 47 studies reporting adherence rates of 80% or higher.

Regarding study design, nearly half of the studies (36/72) used randomized controlled trials (RCTs), followed by 14 single-group studies, 10 observational studies, 9 quasi-experimental studies, 2 case studies, and 1 mixed methods study. Among the 36 RCTs, 12 used a waitlist control (WLC), 10 used treatment as usual (TAU), 8 used an active comparator (AC), 2 used delayed treatment, 2 used a placebo control, and 2 combined WLC with AC. Of the 9 quasi-experimental studies, 2 used WLC, 2 used TAU, 4 used AC, and 1 used a combination of TAU and AC. RCTs are generally considered the strongest design for establishing causal effects [31]. Within this group, studies using an AC provide the most rigorous evidence, as they control for nonspecific factors and reduce biases associated with treatment, whereas those using WLC, TAU, or delayed treatment provide more moderate levels of causal inference [32,33]. In this review, 10 of the 36 RCTs used AC as a comparator (or included AC in combination), and 5 of the 9 quasi-experimental studies also incorporated ACs. Overall, these findings suggest that the evidence base for DMHIs targeting older adults is developing unevenly. Although a substantial proportion of studies used RCT designs, the overall quality of evidence is only moderately strong, as many studies used WLC or TAU designs, which limits the strength of causal inference and interpretation.

### Characteristics of the Interventions and Their Clinical Outcomes

More detailed intervention characteristics are provided in Multimedia Appendix 3. CBT was the most commonly used therapeutic approach (n=32), delivered predominantly via websites (26/32) and frequently tailored to meet the needs of older adults. Other therapeutic modalities included mindfulness (n=2), psychoeducation (n=4), and problem-solving therapy (n=2). Less common approaches included life review therapy (n=1), positive psychology (n=1), acceptance and commitment therapy (n=1), cognitive behavioral stress management (n=1), stress inoculation training (n=1), and cognitive restructuring (n=1).

Regarding the targeted mental health conditions, most interventions focused on depression (n=30), followed by general psychological distress (n=11), psychological distress associated with specific physical health conditions (n=12), anxiety (n=8), and comorbid anxiety and depression (n=8). A small number of studies focused on posttraumatic stress disorder (PTSD; n=2) and grief (n=1).

Regarding technology requirements, 16 of the 72 studies required older adults to own a device, 32 provided devices for participants, and 24 did not report this information. This suggests that at least some studies considered technology access and tried to be digitally inclusive. In terms of technology type, the most frequently used was web-based platforms (n=38), followed by games or exergames (n=7), VR (n=6), mobile apps (n=6), and chatbots (n=4). Less commonly used technologies included robots (n=3), video-only programs (n=3), audio-only programs (n=2), voice assistants (n=1), offline platforms (n=1), and wearable devices (n=1).

Regarding the intervention period, 11 studies lasted fewer than 8 weeks, 31 lasted 8 to 12 weeks, 2 extended beyond 12 weeks, and 28 did not clearly report the program duration. In terms of the number of sessions, 52 studies included fewer than 4 sessions, 11 involved 4 to 8 sessions, and 9 had more than 8 sessions. Overall, most interventions were relatively short in duration, clustering within the under-8-week and 8‐ to 12-week ranges, while only a few extended into longer-term formats. Interventions with fewer sessions also predominated compared with those involving more intensive or extended session series.

Out of 72 papers reviewed, 54 reported positive clinical outcomes. Among these 54 interventions, the majority—33 (61.11%)—used web-based platforms. Other interventions with positive clinical outcomes included 6 games (11.11%), 5 VR (9.26%), 2 mobile apps (3.70%), 3 chatbots (5.56%), 1 robot (1.85%), 1 offline-platform program (1.85%), 1 video-only program (1.85%), 1 audio-only program (1.85%), and 1 voice assistant (1.85%). These findings show that current DMHIs generally demonstrate benefits to users and that positive outcomes are not limited to a single technology type but are instead distributed across multiple technologies.

Among the 54 interventions that reported positive clinical outcomes, 23 targeted depression as a primary or secondary outcome (42.59%), 8 focused on general psychological distress (14.81%), 8 were designed for psychological distress associated with physical health conditions (14.81%), 6 addressed anxiety (11.11%), 6 targeted both anxiety and depression simultaneously (11.11%), 2 focused on PTSD (3.70%), and 1 on grief (1.85%). These findings highlight that DMHIs have demonstrated effectiveness across a wide spectrum of mental health conditions.

### Therapeutic Approaches, Technological Platforms, and Adaptations for Older Adults

Table 3 presents the distribution of DMHIs across different mental health conditions. Web-based platforms were the most commonly used technology across nearly all conditions (total n=38), particularly for depression (n=17), psychological distress with comorbid physical illness (n=7), and comorbid anxiety and depression (n=6). Game-based interventions were the second-most frequently used (total n=7), targeting depression (n=4), and general psychological distress (n=2). These were followed by VR interventions (total n=6), applied across a more diverse set of mental health issues, including depression (n=2), general psychological distress (n=1), psychological distress with comorbid physical illness (n=1), PTSD (n=1), and grief (n=1). Mobile apps (total n=6) were also distributed across multiple conditions, including depression (n=2), general psychological distress (n=1), psychological distress with comorbid physical illness (n=2), and comorbid anxiety and depression (n=1). Other technologies, such as chatbots, voice assistants, audio-only programs, video-only programs, and robots, appeared in smaller numbers.

**Table 3.** Cross-tabulation of technology types and mental health conditions.

	Depression	General psychological distress	Psychological distress with physical condition	Anxiety	Anxiety and depression	Posttraumatic stress disorder	Grief	Total
Web-based	17	4	7	3	6	1	0	38
Game	4	2	0	1	0	0	0	7
VR^a^	2	1	1	0	0	1	1	6
Mobile app	2	1	2	0	1	0	0	6
Chatbot	0	1	1	1	1	0	0	4
Robot	0	2	1	0	0	0	0	3
Video only	0	0	0	3	0	0	0	3
Audio only; offline platform	2	0	0	0	0	0	0	2
Voice assistant	1	0	0	0	0	0	0	1
Offline platform	1	0	0	0	0	0	0	1
Wearable technology	1	0	0	0	0	0	0	1
Total	30	11	12	8	8	2	1	72

a VR: virtual reality.

Across intervention types, web-based programs, particularly those using CBT, were the most developed and commonly used. These interventions were often applied to depression, anxiety, and psychological distress and were most frequently associated with positive clinical outcomes. However, the larger number of web-based CBT studies does not necessarily mean that this modality is superior to other technologies. Mobile apps, VR, chatbots, robots, and voice assistants appeared less frequently, but they suggest a growing diversification of DMHIs for older adults and may offer opportunities for more interactive, creative, and engaging forms of intervention delivery. However, studies and evidence on these technologies remain relatively limited.

In reviewing the designed features of DMHIs, this study also found that older adults, as users, often faced difficulties with navigating online platforms, limited internet skills, and technical issues. Common issues reported by users included poor web interface usability, accessibility barriers, and content perceived as nonengaging [34]. Low digital literacy further complicated the use of mobile apps and web-based interventions, as many older adults struggled with smartphone navigation, small screen sizes, and technical problems, which limited sustained engagement [35]. Some participants also struggled to see the personal relevance of the interventions, resulting in low sustained engagement. They expressed a desire for greater customization and improved mobile functionality to enhance relevance [36].

In response, adjustments were made to better tailor interventions to older adults’ needs. Adaptations to enhance accessibility, usability, and engagement were reported in 23 of the 72 studies, indicating that adaptations tailored to older adults remain relatively uncommon and present substantial opportunities for future development. Among these 23 studies, usability-related design improvements included larger buttons, high-contrast visuals, simplified language, reduced text, and larger fonts in printed training materials [37]. In addition, several interventions used age-specific cases, scenarios, or narratives in educational materials. For instance, the treatment protocol for depression and anxiety was modified by incorporating age-appropriate strategies [38] such as case examples and skill demonstrations tailored specifically for older adults. Similarly, studies also incorporated assistive devices—including sensor pens, tablet holders, and touchscreen tools—to accommodate physical limitations and enhance the accessibility of information and communications technology—communication interventions and information and communications technology—entertainment interventions [39]. Game-based interventions commonly included features such as slower pace, low stimulation, minimal competition, and calming visuals and sounds to match older adults’ physical capacities and processing speeds. For example, 1 study [40] designed cognitive control interventions with these features to reduce anxiety and improve engagement. Moreover, participatory design approaches were commonly used to guide product development to better fit the needs of older adults. For example, 1 study [36] used a “blue-sky” approach—a creative, open-ended design process—to create prototypes focused on meeting the unique needs of older adults.

Specific tailoring strategies varied by technology type (Table 4). Web-based interventions (n=13) most frequently incorporated age-friendly design features, such as large fonts, simplified navigation, and age-relevant case examples. Game-based interventions (n=4), often used in physical exercise programs, reduced difficulty levels and incorporated calming visuals and gentle physical movements, sometimes supported by physical aids. Mobile apps (n=3) offered simplified interfaces and peer support features, with some specifically developed for long-term care settings to address both cognitive and physical health needs. Audio-only and offline platforms (n=2), along with chatbots and robots (n=1 each), incorporated button-based interfaces and multimodal input options (eg, voice and text) to facilitate ease of use and social interaction.

**Table 4.** Tailoring of digital mental health interventions for older adults.

Technology type and target mental health conditions (studies)	Tailoring to the needs of older adults
Web-based (n=13)	
Depression (n=7)	Used age-specific cases or scenarios in educational materials. Applied age-friendly design features to improve usability, such as larger buttons, high-contrast visuals, simplified language, less text, and larger fonts in printed training materials
Anxiety and depression (n=2)	Adjusted content using older adults’ narratives and experiences
General psychological distress (n=2)	Incorporated assistive devices, such as sensor pens, tablet holders, and touchscreen tools, to accommodate physical limitations Used participatory design approaches to tailor product features
Psychological distress with physical conditions (n=1)	Adapted content from interventions previously proven effective for older adults Modified materials to align with older adults’ mental health needs
Anxiety (n=1)	Used age-relevant, real-life scenarios and illustrations Designed therapeutic modules at a slower pace and with less stress to align with users’ processing speed
Game (n=4)	
Depression (n=2)	Adjusted game difficulty to match older adults’ physical capacities Incorporated safety-focused designs considering energy and mobility limitations
Anxiety (n=1)	Included game features such as slower pace, low stimulation, minimal competition, and calming visuals and sounds
General psychological distress (n=1)	Incorporated gentle physical activities and encouraged social interaction through gameplay
Mobile app (n=3)	
Depression (n=2)	Considered care settings (eg, clinical, community, long-term care) in design Simplified interfaces to support older adults with cognitive and communicative limitations
Psychological distress with physical conditions (n=1)	Included peer support features targeting both mental and physical health needs of older adults
Audio only or offline platform (n=2)	
Depression (n=2)	Based on CBT^a^ therapist manuals tailored for older adults Incorporated age-specific examples, larger font sizes, and simplified navigation to improve accessibility
Chatbot (n=1)	
Anxiety and depression (n=1)	Built on a popular platform already familiar to older adults Used button-based interfaces and age-appropriate exercise videos to improve usability and relevance

a CBT: cognitive behavioral therapy.

In Table 4, there are 4 main types of tailoring strategies synthesized from the included studies: interface adaptations, content adaptations, delivery support, and participatory design. Interface adaptations included larger fonts, simplified navigation, high-contrast visuals, button-based interfaces, and reduced text. Content adaptations included age-relevant examples, older adult narratives, slower pacing, and scenarios reflecting later-life experiences. Delivery support included technical assistance, coaching, device provision, and assistive tools to reduce barriers related to digital literacy and physical limitations. Finally, there are also a few interventions that incorporated participatory approaches, in which older adults contributed to intervention development, prototype testing, or usability refinement. All 4 types are important, but they have been developed to different degrees. In existing DMHIs, age-centered design is most often implemented through usability modifications, while deeper forms of participatory development and systematic testing of adaptation strategies remain limited.

Although the reviewed studies did not directly test whether age-related adaptations and specific features themselves contributed to clinical improvements, several features appeared to frequently co-occur with positive engagement with the intervention or clinical outcomes. For example, interventions that provided personalized support, such as coaching or technical assistance, often reported higher engagement and adherence. One example is the Empower@Home program, an intervention whose intellectual property is owned by the Regents of the University of Michigan and which was developed by the author XX and her team [37]. Similarly, programs incorporating social interaction components, such as peer support or group activities, were found to reduce loneliness and improve psychiatric self-management [10]. Age-appropriate content, scenarios, and illustrations were also present in several effective interventions [37,38], while interactive, feedback, and motivational elements appeared in programs with both psychosocial and physical benefits [41].

Taken together, these patterns suggest that age-friendly and age-centered design features may enhance the acceptability and potential benefits of DMHIs for older adults. However, because previous studies did not explicitly test the effects of individual design elements—aside from a few qualitative and mixed methods studies that explored these issues [37,42]—future research should examine these features systematically to clarify their specific contributions to engagement and outcomes.

## Discussion

### Summary of Study Findings

This scoping review provides an overview of the current development of DMHIs targeting older adults. These DMHI programs have used a range of technologies, such as web-based platforms, mobile apps, VR, and chatbots. In recent years, the rapid development of AI has offered new possibilities for personalizing and adapting DMHIs even further. However, we identified few studies that used AI. This may partly reflect the limitations of not including specific AI-related terms in the literature search and our general focus on digital interventions, as well as the typical lag between recent technological innovation—such as the large language model boom in industry—its adoption in real-world products, and subsequent academic work [43]. As emerging technologies like AI continue to advance, future research on DMHIs for older adults should incorporate the evolution and upgrading of technology and examine how these exciting changes shape intervention design, delivery, and evaluation, as well as how emerging technologies can be integrated ethically and effectively into DMHIs targeting older adults in real-world practice [44].

This review also informs us that, beyond technological innovation, tailoring products to older adult users’ needs and making products aging-appropriate are also important. The active participation of older adults in both the design and evaluation stages can help make products accessible, usable, and age-friendly. Evidence suggests that involving older adults throughout the design process helps ensure that interventions align with users’ needs and preferences, which can improve both engagement and effectiveness [45,46]. However, this review found that only a few studies applied a co-design approach to developing or evaluating DMHIs for older adults—for example, a co-design workshop to test a chatbot prototype [47] and a brainstorming session on mobile app design [36]. Future DMHI design or adaptation should consider actively engaging older adults across all stages to help ensure that interventions are culturally and contextually relevant.

Regarding age-adaptation design, in this review, 31.94% (n=23/72) of the included studies developed or tested interventions that incorporated older adult–centered features to enhance usability and engagement, which shows that current DMHIs with older adult–centered design are underdeveloped to some extent. One possible reason is that many interventions are originally designed for the general population and then adapted for older adults during the launch or usability testing stage to expand the user market [48,49]. This indicates an existing gap between the growing realization of the importance of older adults as target users and the development of underlying design principles for age-centered tailoring in intervention development or improvement.

Also, prior research has highlighted the unique challenges that older adults face when using DMHIs, including usability barriers, accessibility issues, and difficulties integrating technology into daily routines [50]. Many of the interventions in these 23 studies incorporated age-friendly design features, such as larger buttons, high-contrast visuals, simplified language, and larger fonts to accommodate physical and cognitive limitations, which aligns with the aim of overcoming the challenges mentioned above. These 23 studies also inform us that most age-related adaptations are simple and focus on improving ease of use and accessibility tailored to older adults, which also echoes previous studies that caution that complex or visually “fancy” interfaces do not necessarily improve outcomes; rather, simple and accessible designs tend to be more usable and acceptable [14,50].

Regarding future research and practice, the review establishes multiple essential research paths that will help scientists and practitioners enhance their understanding of DMHIs for older adults. First, this study revealed that while nearly half of the evaluation studies used RCT designs, most studies used waitlist or TAU control groups as their comparison conditions. These research designs may limit the ability to establish clear cause-and-effect relationships and may also produce exaggerated treatment outcomes [31]. Future research should strengthen methodological rigor by using RCTs with active comparison groups and longer follow-up periods. In addition, future evaluations should not only assess clinical effectiveness but also examine whether interventions are usable, accessible, and appropriate for older adults in real-world settings.

Second, the studies presented in Tables2  3 demonstrate that most investigations took place in high-income countries, particularly Australia and the United States, while minority groups and people from low- and middle-income countries remained underrepresented. This limited representation restricts the generalizability of the findings and may contribute to digital health disparities, as interventions developed in resource-rich settings may not reflect the needs, preferences, language backgrounds, cultural contexts, or technology access barriers of diverse older adult populations. From a participatory design perspective, the development of inclusive DMHIs requires researchers to actively involve diverse older adults and community stakeholders throughout the design, adaptation, and evaluation process [48]. Future research should, therefore, expand studies in low- and middle-income countries and among diverse older adult populations to better address mental health disparities [51].

Third, this review found that only about one-third of studies incorporated usability adaptations or age-centered design strategies, while most did not sufficiently address digital literacy, technology accessibility, or user interface challenges faced by older adults. From a universal design perspective, DMHIs should aim to reduce barriers related to accessibility, usability, health literacy, sensory changes, and technology confidence, while avoiding the assumption that older adults are a homogeneous user group [52]. Gerontechnology frameworks similarly emphasize the importance of aligning technological design with the physical, cognitive, emotional, and social dimensions of aging [53]. Therefore, future interventions should move beyond simply adapting existing digital tools for older adults and instead use more theory-informed and participatory approaches to age-centered design.

### Limitations

This scoping review has several methodological limitations that should be considered when interpreting the findings. First, our search strategy was intentionally broad to capture the wide range of DMHIs. While this approach allowed us to map the landscape of existing interventions, it may have missed studies in niche areas that use more specialized terminology. For example, we did not include more specific terms such as machine learning, deep learning, natural language processing, large language model, and generative AI. Therefore, our review may have limited the inclusion of AI-powered interventions. As AI-enabled DMHIs may differ from traditional DMHIs in terms of user interaction, data use, data safety, and ethical risks, the lack of inclusion of AI-enabled DMHIs may limit our ability to capture important trends in this rapidly emerging area of DMHI research. Second, as a scoping review, our goal was to describe and categorize the types of technologies, intervention designs, and adaptations rather than to evaluate intervention efficacy quantitatively. Because we included a wide variety of study designs, intervention types, and outcomes, our ability to compare or quantitatively synthesize the study findings was limited. Another limitation is that we did not conduct a formal quality appraisal or risk-of-bias assessment in this study. While formal quality appraisal is not always required for scoping reviews, the absence of such an assessment limits our ability to evaluate the methodological rigor of each study. Because studies with different designs, samples, comparison conditions, and follow-up periods were synthesized together, this study provides a descriptive overview that scopes and maps the current evidence rather than offering evidence of intervention effectiveness or study quality across all included studies. Future reviews with a narrower scope could conduct formal quality appraisal and quantitative synthesis to better assess intervention effectiveness. Third, the quality of individual studies was not systematically assessed. Judging by the range of study designs included, many interventions would benefit from more rigorous evaluation methods, such as RCTs with adequate sample sizes and follow-up periods. In addition, we only included studies published in English, which likely explains why most included studies were conducted in Western or Global North countries. Finally, we primarily included peer-reviewed journal studies and may have missed gray literature, potentially leading to publication bias.

### Conclusions

This review examined the increasing yet unequal growth of DMHIs, which provide services to adults who are 50 years or older. The 72 studies demonstrated that DMHIs using web-based interventions and other digital tools, including games, VR, mobile apps, chatbots, and robots, produced positive results for the treatment of depression, anxiety, and psychological distress in older adults. The current evidence supporting DMHIs for older adults remains moderate because most studies lack active comparison groups and fail to demonstrate real-world implementation. The research found that age-related adaptations and co-design methods to address age-specific design requirements and usage remained limited throughout the study. Future research needs to establish trials with active comparison groups and extended observation periods, as well as conduct studies on DMHI implementation in regular health care services and community-based environments while including diverse older adult groups and testing user-friendly features with technology training and access support to develop better DMHIs for older adults.

## Supplementary material

10.2196/92542 Multimedia Appendix 1 Search terms used for article retrieval in databases.

10.2196/92542 Multimedia Appendix 2 Descriptive analysis of the 72 included studies.

10.2196/92542 Multimedia Appendix 3 Characteristics of interventions in the 72 included studies.

10.2196/92542 Checklist 1 PRISMA-ScR checklist.

## Abbreviations

AC: active comparator
AI: artificial intelligence
CBT: cognitive behavioral therapy
DMHI: digital mental health intervention
PICO: population, intervention, comparator, and outcome
PRISMA: Preferred Reporting Items for Systematic Reviews and Meta-Analyses
PRISMA-ScR: Preferred Reporting Items for Systematic Reviews and Meta-Analyses extension for Scoping Reviews
PTSD: posttraumatic stress disorder
RCT: randomized controlled trials
TAU: treatment as usual
VR: virtual reality
WLC: waitlist control
