# Supplementary material for: Landscape, Evidence, Gaps, and Opportunities in Digital Mental Health Interventions for Older Adults: Scoping Review
Source: Interact J Med Res. 2026 Jul 6;15:e92542. doi: 10.2196/92542 (PMC13335415; doi:10.2196/92542)
Supplement: Multimedia Appendix 1 [file ijmr-v15-e92542-s001.docx]

Supplemental Appendix 1: Search Terms and Results

We restricted the selection to works in English and those that are peer-reviewed. Publications were restricted to the years 2000 to 2025. Initial screening was conducted in June 2022 and second round of screening was conducted in February 2025. The results were then downloaded for abstract and title screening and full-text screening.

| Database | Search Strings | Dates Searched | Results |
| --- | --- | --- | --- |
| PsychINFO | String1: ( (MA “Mobile Applications” OR  mobile apps OR chatbot OR conversational agent OR artificial intelligence OR video game) AND (AB Mental Health OR AB Anxiety OR AB Depression OR AB Psychotherapy) ) NOT AG Adolescence (13-17 yrs) NOT AG Young Adulthood (18-29 yrs) NOT AG Thirties (30-39 yrs)  String2: ( (SU "Computer" OR SU "Digital" OR SU “technology” OR SU “internet” OR SU “Mobile health” OR SU “mHealth” OR SU “Online Therapy”) AND (AB depression OR AB depressed OR AB Anxiety OR AB Mental Health) ) NOT AG Adolescence (13-17 yrs) NOT AG Young Adulthood (18-29 yrs) NOT AG Thirties (30-39 yrs)  String3: ( (“Computer-assisted therapy” OR “technology-assisted” OR “web-based” OR “internet-based” OR “computer-based” or “technology-based” OR “online intervention” OR “digital interventions” OR computerized therapy) AND (AB depression OR AB depressed OR AB Anxiety OR AB “Mental Health” OR AB Psychosocial OR AB Psychotherapy) ) NOT AG Adolescence (13-17 yrs) NOT AG Young Adulthood (18-29 yrs) NOT AG Thirties (30-39 yrs)  Specify limiters: Check “Peer reviewed”, “English”, “Published date Jan 2000”; Leave end date blank’default; Select [Middle Age (40-64 yrs), Aged (65 yrs & older), Very Old (85 yrs & older)](https://rss.ebscohost.com/AlertSyndicationService/Syndication.asmx/GetFeed?guid=9077826) | June 16, 2022 | 699 |
|  | String1: ( (MA “Mobile Applications” OR  mobile apps OR chatbot OR conversational agent OR artificial intelligence OR video game) AND (AB Mental Health OR AB Anxiety OR AB Depression OR AB Psychotherapy) ) NOT AG Adolescence (13-17 yrs) NOT AG Young Adulthood (18-29 yrs) NOT AG Thirties (30-39 yrs)  String2: ( (SU "Computer" OR SU "Digital" OR SU “technology” OR SU “internet” OR SU “Mobile health” OR SU “mHealth” OR SU “Online Therapy”) AND (AB depression OR AB depressed OR AB Anxiety OR AB Mental Health) ) NOT AG Adolescence (13-17 yrs) NOT AG Young Adulthood (18-29 yrs) NOT AG Thirties (30-39 yrs)  String3: ( (“Computer-assisted therapy” OR “technology-assisted” OR “web-based” OR “internet-based” OR “computer-based” or “technology-based” OR “online intervention” OR “digital interventions” OR computerized therapy) AND (AB depression OR AB depressed OR AB Anxiety OR AB “Mental Health” OR AB Psychosocial OR AB Psychotherapy) ) NOT AG Adolescence (13-17 yrs) NOT AG Young Adulthood (18-29 yrs) NOT AG Thirties (30-39 yrs)  Specify limiters: Check “Peer reviewed”, “English”, “Published date Jan 2022”; Leave end date blank’default; Select [Middle Age (40-64 yrs), Aged (65 yrs & older), Very Old (85 yrs & older)](https://rss.ebscohost.com/AlertSyndicationService/Syndication.asmx/GetFeed?guid=9077826) | February 13, 2025 | 276 |
| PubMed | ("Computer" OR "Digital" OR “technology” OR “internet” OR “Mobile health” OR “mHealth” OR “Online Therapy” OR “Computer assisted therapy” OR “technology-assisted”  OR “web-based” OR “internet-based” OR “computer-based” or “technology-based” OR “online intervention” OR “digital intervention” OR computerized therapy OR “Mobile Applications” OR mobile apps OR chatbot OR conversational agent OR artificial intelligence OR video game) AND (depression OR depressed OR Anxiety OR Mental Health) AND (Psychotherapy or Intervention OR treatment) AND (older adults OR elderly) | June 12, 2022 | 382 |
|  | ("Computer" OR "Digital" OR “technology” OR “internet” OR “Mobile health” OR “mHealth” OR “Online Therapy” OR “Computer assisted therapy” OR “technology-assisted”  OR “web-based” OR “internet-based” OR “computer-based” or “technology-based” OR “online intervention” OR “digital intervention” OR computerized therapy OR “Mobile Applications” OR mobile apps OR chatbot OR conversational agent OR artificial intelligence OR video game) AND (depression OR depressed OR Anxiety OR Mental Health) AND (Psychotherapy or Intervention OR treatment) AND (older adults OR elderly) | February 11, 2025 | 291 |
| Scopus | TITLE-ABS-KEY ( ( “Computer assisted therapy” OR “Technology assisted”  OR “Web-based” OR “Internet-based” OR “Computer-based” OR “Technology-based” OR “Online intervention” OR “Digital interventions” OR “Computerized therapy” OR “Computer” OR “digital” or “internet” or “Mobile Health” or “mhealth” or “online therapy” or “Mobile applications” or “mobile app” or “chatbot” or “conversational agent” or “artificial intelligence” or “video games”)  AND  ( “Depression” OR “Depressed”  OR “Anxiety” OR mental health) AND (“Psychotherapy” or “Intervention” OR “Treatment”) AND  ( "Older adults"  OR  "Elderly" ) )  AND  PUBYEAR  >  2000 | June 14, 2022 | 1124 |
|  | TITLE-ABS-KEY ( ( "Computer assisted therapy" OR "Technology assisted" OR "Web-based" OR "Internet-based" OR "Computer-based" OR "Technology-based" OR "Online intervention" OR "Digital interventions" OR "Computerized therapy" OR "Computer" OR "digital" OR "internet" OR "Mobile Health" OR "mhealth" OR "online therapy" OR "Mobile applications" OR "mobile app" OR "chatbot" OR "conversational agent" OR "artificial intelligence" OR "video games" ) AND ( "Depression" OR "Depressed" OR "Anxiety" OR mental AND health ) AND ( "Psychotherapy" OR "Intervention" OR "Treatment" ) AND ( "Older adults" OR "Elderly" ) ) AND PUBYEAR > 2021 AND ( LIMIT-TO ( LANGUAGE , "English" ) ) | February 11, 2025 | 690 |
| Web of Science | (((TI=(( ( “Computer assisted therapy” OR “Technology assisted”  OR “Web-based” OR “Internet-based” OR “Computer-based” OR “Technology-based” OR “Online intervention” OR “Digital interventions” OR “Computerized therapy” OR  "Computer" OR "Digital" OR “Technology” OR “Internet” OR “Mobile health” OR “mHealth” OR “Online Therapy” or "Mobile Applications"  OR “Mobile app” OR "Chatbot"  OR  "Conversational agent"  OR  "Artificial Intelligence"  OR  "Video Game" or “Virtual assistant” )  AND  ( “Depression” OR “Depressed”  OR “Anxiety” OR “Mental Health” or “Mental illness” ) AND (“Psychotherapy” or “Intervention” OR “Treatment”) AND  ( "Older adults"  OR  "Elderly" )))) OR AB=(( ( “Computer assisted therapy” OR “Technology assisted”  OR “Web-based” OR “Internet-based” OR “Computer-based” OR “Technology-based” OR “Online intervention” OR “Digital interventions” OR “Computerized therapy” OR  "Computer" OR "Digital" OR “Technology” OR “Internet” OR “Mobile health” OR “mHealth” OR “Online Therapy” or "Mobile Applications"  OR “Mobile app” OR "Chatbot"  OR  "Conversational agent"  OR  "Artificial Intelligence"  OR  "Video Game" or “Virtual assistant” )  AND  ( “Depression” OR “Depressed”  OR “Anxiety” OR “Mental Health” or “Mental illness” ) AND (“Psychotherapy” or “Intervention” OR “Treatment”) AND  ( "Older adults"  OR  "Elderly" )))) OR KP=(( ( “Computer assisted therapy” OR “Technology assisted”  OR “Web-based” OR “Internet-based” OR “Computer-based” OR “Technology-based” OR “Online intervention” OR “Digital interventions” OR “Computerized therapy” OR  "Computer" OR "Digital" OR “Technology” OR “Internet” OR “Mobile health” OR “mHealth” OR “Online Therapy” or "Mobile Applications"  OR “Mobile app” OR "Chatbot"  OR  "Conversational agent"  OR  "Artificial Intelligence"  OR  "Video Game" or “Virtual assistant” )  AND  ( “Depression” OR “Depressed”  OR “Anxiety” OR “Mental Health” or “Mental illness” ) AND (“Psychotherapy” or “Intervention” OR “Treatment”) AND  ( "Older adults"  OR  "Elderly" )))) AND PY=(2000-2022) | June 14, 2022 | 329 |
|  | (((TI=(( ( “Computer assisted therapy” OR “Technology assisted”  OR “Web-based” OR “Internet-based” OR “Computer-based” OR “Technology-based” OR “Online intervention” OR “Digital interventions” OR “Computerized therapy” OR  "Computer" OR "Digital" OR “Technology” OR “Internet” OR “Mobile health” OR “mHealth” OR “Online Therapy” or "Mobile Applications"  OR “Mobile app” OR "Chatbot"  OR  "Conversational agent"  OR  "Artificial Intelligence"  OR  "Video Game" or “Virtual assistant” )  AND  ( “Depression” OR “Depressed”  OR “Anxiety” OR “Mental Health” or “Mental illness” ) AND (“Psychotherapy” or “Intervention” OR “Treatment”) AND  ( "Older adults"  OR  "Elderly" )))) OR AB=(( ( “Computer assisted therapy” OR “Technology assisted”  OR “Web-based” OR “Internet-based” OR “Computer-based” OR “Technology-based” OR “Online intervention” OR “Digital interventions” OR “Computerized therapy” OR  "Computer" OR "Digital" OR “Technology” OR “Internet” OR “Mobile health” OR “mHealth” OR “Online Therapy” or "Mobile Applications"  OR “Mobile app” OR "Chatbot"  OR  "Conversational agent"  OR  "Artificial Intelligence"  OR  "Video Game" or “Virtual assistant” )  AND  ( “Depression” OR “Depressed”  OR “Anxiety” OR “Mental Health” or “Mental illness” ) AND (“Psychotherapy” or “Intervention” OR “Treatment”) AND  ( "Older adults"  OR  "Elderly" )))) OR KP=(( ( “Computer assisted therapy” OR “Technology assisted”  OR “Web-based” OR “Internet-based” OR “Computer-based” OR “Technology-based” OR “Online intervention” OR “Digital interventions” OR “Computerized therapy” OR  "Computer" OR "Digital" OR “Technology” OR “Internet” OR “Mobile health” OR “mHealth” OR “Online Therapy” or "Mobile Applications"  OR “Mobile app” OR "Chatbot"  OR  "Conversational agent"  OR  "Artificial Intelligence"  OR  "Video Game" or “Virtual assistant” )  AND  ( “Depression” OR “Depressed”  OR “Anxiety” OR “Mental Health” or “Mental illness” ) AND (“Psychotherapy” or “Intervention” OR “Treatment”) AND  ( "Older adults"  OR  "Elderly" )))) AND PY=(2022-2025) | February 11, 2025 | 240 |
| AgeLine | String for Abstract Search: (“Online" OR” computer-based” OR “technology based” OR web based OR “internet” Or “computer-assisted” or “chatbot" or “conversational agent” or “virtual assistant” or “video game” or “online intervention” or “digital intervention” or “computerized therapy”)  AND ("depression" OR "Anxiety" OR "mental health" OR "mental illness" OR "psychiatric disease" OR "psychiatric illness") and (“Intervention” or “treatment” or “therapy”)  String for Subject Search: (SU “Mental Health” OR  “Anxiety” OR “Depression” OR “Geriatric Psychiatry” OR “Mood disorders”) AND (SU “Cognitive Behavioral Therapy” OR “Therapy” OR “Psychotherapy” OR “Treatment” OR “Psychiatric Services” ) AND (AB “internet” OR “Online” Or “Digital” OR “computer” OR “computerized” or “mhealth” Or “mobile app”)  Set Publication Date as: 2000-01-01 to Present | June 12, 2022 | 103 |
|  | String for Abstract Search: (“Online" OR” computer-based” OR “technology based” OR web based OR “internet” Or “computer-assisted” or “chatbot" or “conversational agent” or “virtual assistant” or “video game” or “online intervention” or “digital intervention” or “computerized therapy”)  AND ("depression" OR "Anxiety" OR "mental health" OR "mental illness" OR "psychiatric disease" OR "psychiatric illness") and (“Intervention” or “treatment” or “therapy”)  String for Subject Search: (SU “Mental Health” OR  “Anxiety” OR “Depression” OR “Geriatric Psychiatry” OR “Mood disorders”) AND (SU “Cognitive Behavioral Therapy” OR “Therapy” OR “Psychotherapy” OR “Treatment” OR “Psychiatric Services” ) AND (AB “internet” OR “Online” Or “Digital” OR “computer” OR “computerized” or “mhealth” Or “mobile app”)  Set Publication date as: 2022-01-01 to Present | February 13, 2025 | 114 |
