# Supplementary material for: Landscape, Evidence, Gaps, and Opportunities in Digital Mental Health Interventions for Older Adults: Scoping Review
Source: Interact J Med Res. 2026 Jul 6;15:e92542. doi: 10.2196/92542 (PMC13335415; doi:10.2196/92542)
Supplement: Multimedia Appendix 2 [file ijmr-v15-e92542-s002.docx]

Table1 Study Design and sample

| Authors | Country | Study Design | Comparator | Follow up | Recruitment Setting | Sample size | Sample age mean(sd) | % Female |
| --- | --- | --- | --- | --- | --- | --- | --- | --- |
| Knowles et al., 2017 | USA | Quasi-experimental | AC | Yes (2 months) | Newspaper obituaries | 30 | 67.20 (10.73) | 70 |
| Dear et al., 2015 | Australia | RCT | WLC | Yes (3 months & 12 months) | Website | 72 | Treatment: 65.39 (4.68); Control: 65.51 (5.81) | Treatment: 67; Control: 54 |
| McMurchie et al., 2013 | Scotland | Quasi-experimental | TAU | Yes (3 months) | Older People Community Mental Health Teams (OPCMHT) | 53 | Treatment: 71.58 (4.43); Control: 75.55 (6.27) | Treatment: 75.8; Control: 70 |
| Gould et al., 2017 | USA | Observational study | N/A | Not reported | Advertisements in VA medical centers, community-based outpatient VA clinics, Vet Center, on Craigslist, VA medical center Facebook page | 20 | 69.50 (7.30) | 15 |
| Xiang et al., 2021 | USA | Observational study | N/A | Not reported | Community advertisements and referrals | 21 | 76.00 (9.10) | 81 |
| Spek et al., 2007 | The Netherlands | RCT | WLC | Yes (1 year) | Advertisements in free regional newspapers, and by personal letters sent by the Municipal Health Care Service of the city of Eindhoven | 301 | 55.00 (4.68) | 63.24 |
| Staples et al., 2016 | Australia | Quasi-experimental | AC | Yes (3 months) | Clinic website or eCentreclinic | 949 | 65.70 (5.10) | 59 |
| Preschl et al., 2012 | Switzerland | RCT | WLC | Yes (3 months) | Advertisement | 36 | 72.50 (4.50) | 15 |
| Chao et al., 2015 | USA | Quasi-experimental | AC | Not reported | Assisted living facilities | 32 | 85.19 (6.47) | 75.8 |
| Read et al., 2021 | Australia | RCT | TAU | Yes (24 months) | Advertising from social media | 302 | 73.00 (5.90) | 70 |
| Villani et al., 2018 | Italy | RCT | TAU | Yes (3 months) | Through direct patient contact by oncologists at hospitals | 29 | 62.76 (6.19) | 100 |
| Fields et al., 2021 | USA | Single group | N/A | Not reported | Residential care | 15 | 85.80 (4.47) | 26.7 |
| Benda et al., 2020 | USA | Single group | N/A | Not reported | Senior center | 15 | Not reported | 53.3 |
| Bond et al., 2010 | USA | RCT | TAU | Yes (6 months) | Flyers, provider referral, or letters | 62 | Treatment: 66.2 (5.7); Control: 68.2 (6.2) | Treatment: 42; Control: 48 |
| Bennion et al., 2020 | UK | RCT | AC | Yes (2 weeks) | Advertised over the Web via U3A websites and offline via recruitment posters placed within U3A meeting places | 112 | 69.21 (6.76) | 73.2 |
| Shaunfield et al., 2014 | USA | Single group | N/A | Not reported | Assisted living center | 21 | 85.10 | 85.71 |
| O'moore et al., 2018 | Australia | RCT | TAU | Yes (3 months) | Health care organizations | 69 | 62.00 (7.07) | 80 |
| Anguera et al., 2017 | USA | RCT | TAU | Yes (4 weeks post intervention) | San Francisco Area | 22 | 68.00 (6.30) | 72.73 |
| Yanez et al., 2015 | USA | RCT | AC | Yes (6 months) | Robert H. Lurie Comprehensive Cancer Center of Northwestern University and the Jesse Brown VA Medical Center in Chicago. | 74 | 68.84 (9.23) | 0 |
| Xiang et al., 2020 | USA | Single group | N/A | Not reported | Local home care company, senior apartment buildings, and Meals-on-Wheels in Southeast Michigan. | 26 | 76.40 (9.20) | 69.2 |
| Ying et al., 2021 | China | Single group | N/A | Yes (1 month) | Nursing home | 127 | 73.39 (7.37) | 68.5 |
| Rosenberg et al., 2010 | USA | Single group | N/A | Yes (20-24 weeks) | Senior community centers and retirement communities | 19 | 78.70 (8.70) | 68.42 |
| Nilsson et al., 2021 | Sweden | RCT | TAU | Yes (4-6 weeks after surgery) | Hospital | 120 | 73.00 | 13 |
| Read et al., 2020 | Australia | RCT | TAU | Yes (3 & 6 months) | Participant pool, clinics, health organizations | 302 | 73.00 (5.90) | 70.2 |
| Šabanović et al., 2015 | USA | Observational study | N/A | Not reported | Identified by Outpatient healthcare provider | 5 | Not reported | 20 |
| Easton et al., 2019 | UK | Observational study | N/A | Not reported | Identified through the local British Lung Foundation Breathe Easy support group | Workshop1: 5; Workshop2: 4 | Not reported | Workshop1: 60; Workshop2: 50 |
| Henrique et al., 2021 | Brazil | Observational study | N/A | Not reported | Basic Health Unit (UBS in Portuguese) | 31 | 71.35 (6.50) | 74.1935 |
| Shah et al., 2018 | USA | RCT | DT | Not reported | Primary care sites, senior centers, senior residential facilities, and churches | 51 | 63.69 (7.17) | 84.3 |
| Fortuna et al., 2018 | USA | Observational study | N/A | Not reported | Home health agency | 8 | 68.80 (4.90) | 87.5 |
| Morthland et al., 2020 | USA | RCT | DT | Not reported | Healthcare settings | 51 | 63.69 (7.17) | 84.3 |
| Knaevelsrud et al., 2017 | Germany | RCT | WLC | Yes (treatment group at 3&6&12month follow-ups; wlc: 6-week). | Primary care practices, referrals from clinicians, and radio, newspaper, and an open access Web site | 94 | 71.40 (4.70) | 64.9 |
| Li et al., 2018 | Singapore | RCT | AC | Not reported | Senior activity centers and community clubs | 102 | 71.40 (7.87) | 63.7 |
| Chao et al., 2014 | USA | Single group | N/A | Not reported | Assisted living facilities | 7 | 86.00 (5.00) | 71.43 |
| Fortuna et al., 2018 | USA | Single group | N/A | Not reported | Identified and recruited by clinical team leader and case managers | 10 | 68.80 (4.90) | 87.5 |
| Silfvernagel et al., 2018 | Sweden | RCT | AC | Yes (1 year) | Advertisements in a Swedish newspaper | 66 | 66.10 (4.15) | 75.8 |
| Dear et al., 2013 | Australia | Single group | N/A | Yes (3 months) | Through a clinical research website | 20 | 63.40 (5.08) | 65 |
| Wada et al., 2006 | Japan | Single group | N/A | Not reported | Health service facility | 14 | 88.2 (6) | 100 |
| Schneider et al., 2003 | USA | RCT | TAU | Not reported | Outpatient cancer center | 16 | 57.70 (6.80) | 100 |
| Jones et al., 2016 | Canada | RCT | WLC | Yes (1 month) | Via newspapers, newsletters, radio announcements, online advertisements, posters, and letters sent to physicians and psychiatrists. | 46 | 65.13 (4.24) | 86.95 |
| Muroi et al., 2020 | China | RCT | PC | Not reported | Not reported | 42 | 67.79 | 30.95 |
| Zou et al., 2012 | Australia | Single group | N/A | Yes (3 months) | Applicants applied online | 22 | 66.00 (4.60) | 68 |
| Chen et al., 2020 | USA | Quasi-experimental | WLC | Not reported | Recruited via research  Registries, online and community advertisements and  clinic referrals. | 47 | 69.60 (4.10) | 68.1 |
| Hwang et al., 2021 | USA | Single group | N/A | Yes (12 months) | Senior centers, churches, other community groups, and Aging and Disability Resource Centers. | 197 | 76.26 (7.38) | 73.6 |
| Orr et al., 2020 | USA | Case studies | N/A | Not reported | Primary care clinics at the University of Arkansas for Medical Sciences (UAMS) | 3 | 63.67 | 66.6 |
| Dear et al., 2015 | Australia | RCT | WLC | Yes (3 months) | Applied online to participate via an established website (www.ecentreclinic.org) | 47 | Anxiety Trial: 65.3 (5.33); Depression Trial: 66.6 (3.71) | Anxiety Trial: 48.1; Depression Trial: 70.0 |
| Li et al., 2016 | Singapore | RCT | AC | Not reported | Senior activity centers and community clubs | 49 | 71.12 (8.67) | 59.2 |
| Similä et al., 2018 | Finland | Observational study | N/A | Not reported | Circle of Friends group and family caregivers' peer support group offered by the communal senior services | 7 | 73.00 (7.00) | 100 |
| Tomasino et al., 2017 | USA | Quasi-experimental | WLC | Yes (8 weeks) | Clinical research registries, online and community advertisements, and clinic referral | 47 | 69.40 (4.10) | 68.1 |
| Chiu & Wu, 2019 | Taiwan | RCT | TAU | Not reported | Community-based long-term care facilities | 54 | 73.00 (11.40) | 50 |
| Wahbeh, 2018 | USA | RCT | WLC | Not reported | Institute of Noetic Sciences (IONS) community network, online listservs and research opportunities postings, flyers posted at older adult community locations; and outreach to older adult housing and social groups | 50 | 64.80 (6.20) | 81 |
| Spek et al., 2008 | The Netherlands | RCT | WLC | Yes (1 year) | Send letter to recruit | 301 | 55.00 (4.60) | 63.12 |
| Proyer et al., 2014 | Switzerland | RCT | PC | Yes (1&3&6 months) | Participants registered at a free website affiliated | 163 | 55.58 (5.16) | 100 |
| Gamito et al., 2010 | Portugal | RCT | WLC | Not reported | Had experience of participating in the Portuguese colonial war between 1963 and 1970 in Africa | 10 | 63.50 (4.43) | 0 |
| Lappalainen et al., 2022 | Finland | Quasi-experimental | TAU | Not reported | Advertisements in local newspapers, recruited from rehabilitation courses and family caregiver associations | 149 | 72.90 (6.10) | 80.5 |
| Titov et al., 2016 | Australia | RCT | AC | Yes (3 months) | Recruited via a specialist research unit (www. ecentreclinic.org) | 433 | 66.00 (4.70) | 64 |
| Gould et al., 2019 | USA | RCT | WLC | Yes (8 weeks) | Recruited through flyers, internet advertisements, community presentations, and Veterans Affairs and Stanford provider referrals as well as through contacting past participants of other research studies | 40 | 68.93 (7.13) | 60 |
| Titov et al., 2015 | Australia | RCT | WLC | Yes (3&12 months) | Recruited via website | 54 | Treatment: 64.52 (2.58); Control: 66.16 (3.8) | Treatment: 81.5; Control: 64 |
| Xiang et al., 2024 | United States | RCT | WLC | Yes (10 weeks) | Web-based research registries, social media advertisements, and community  agency referrals | 70 | Treatment: 69.8 (5.4); Control: 67.9 (5.8) | Treatment: 77; Control: 85 |
| Kong et al., 2024 | China | RCT | WLC+AC | Yes (6 months &12months) | Institutional long-term care | 354 | 71.6 (7.80) | 68.3 |
| Danieli et al., 2022 | Italy | RCT | WLC+AC | Yes (3 months) | Social Media | 45 | 55.58 (5.08) | 78 |
| Chou et al., 2024 | Taiwan | Observational study | N/A | Yes (within half a year post intervention) | Psychiatric outpatient department of hospital | 35 | 65.21 (7.51) | 74 |
| Nordgren et al., 2024 | Sweden | RCT | AC | Yes (12 months) | Online Platform | 101 | 71.9 (4.4) | 70 |
| Primavera et al., 2024 | Italy | RCT | TAU | Not reported | Consultation and Psychosomatic Psychiatry Unit | 21 | Treatment: 61.5 (3.6); Control: 61.3 (2.5) | Treatment: 78.6; Control: 66.7 |
| Lappalainen et al., 2022 | Finland | Quasi-experimental | TAU+AC | Not reported | Web-based and in-person support | 149 | 72.9 (6.1) | 80.5 |
| Xiang et al., 2024 | USA | Qualitative study | N/A | Not reported | Home-based (remote, Michigan) | 148 | 66 (8.01) | 78 |
| Fan et al., 2022 | Taiwan | Quasi-experimental | AC | Not reported | Community elderly service centers | 62 | Treatment: 70.94 (5); Control: 69.83 (3.8) | Treatment: 75; Control: 90 |
| Borghouts et al., 2022 | USA | Mixed-methods study | N/A | Yes (8 months) | Home-based, supported by county services | 30 | 72 (7.8) | 93 |
| Xiang et al., 2023 | USA | Observational study | N/A | Yes (10 weeks) | Home-based; participants recruited via community partners | 10 | 71.4 (6.4) | 60 |
| Xiang et al., 2024 | USA | Single Group | N/A | Yes (10 weeks) | Home-based, Michigan | 103 | 63.7 (8.59) | 78.6 |
| Giordano et al., 2024 | Germany | Single Group | N/A | Yes (3 months) | Long-term care homes | 40 | Treatment: 79.93 (11.96); Control: 81.5(9.9) | Treatment:  78.57; Control: 50 |
| Gould et al., 2024 | USA | RCT | AC | Yes (4 weeks and 8 weeks) | Home-based, video/DVD + phone coaching | 56 | 71.4 (6.2) | 12 |
| Park & Kim, 2022 | South Korea | Observational study | N/A | Yes (2 months) | Home-based (AI Care Project) | 291 | 76.69 (5.83) | 73.9 |
