# Supplementary material for: Landscape, Evidence, Gaps, and Opportunities in Digital Mental Health Interventions for Older Adults: Scoping Review
Source: Interact J Med Res. 2026 Jul 6;15:e92542. doi: 10.2196/92542 (PMC13335415; doi:10.2196/92542)
Supplement: Multimedia Appendix 3 [file ijmr-v15-e92542-s003.docx]

Table2 Program Characteristics

| Authors | MH conditions | Tech requirement | Tech involved | Treatment type | Length | Completion rate | Designing for Tailoring to older adults | Main Findings |
| --- | --- | --- | --- | --- | --- | --- | --- | --- |
| Knowles et al., 2017 | Grief | Yes | VR | Psychoeducation | 16 sessions in 8 weeks | 28/30 | N/A | 1) Both the virtual reality (VR) support group and the active control grief education website were feasible and acceptable to participants.  2) Both the VR support group and the grief education website group showed significant improvements across a range of outcomes including grief severity, grief cognitions, loneliness, yearning, perceived stress, and global sleep quality. Only participants in the VR support group showed a significant improvement in symptoms of depression over time. |
| Dear et al., 2015 | Anxiety | Not reported | Web-based | CBT | 8 weeks | 61/72 | N/A | 1) iCBT was effective in significantly reducing symptoms of anxiety and depression among older adults aged 60 and above. Participants in the treatment group showed significantly lower scores on measures of anxiety and depression compared to the waitlist control group at post-treatment, with these improvements maintained at 3-month and 12-month follow-up.  2) The iCBT program was well-received by participants, with a high completion rate and positive ratings for satisfaction, indicating that iCBT is an acceptable form of treatment for this population. |
| McMurchie et al., 2013 | Anxiety and Depression | Not reported | Web-based | CBT | 8 weeks | 24/33 | N/A | 1) The study demonstrated that the computerized cognitive behavioral therapy program, Beating the Blues (BTB), was both acceptable and effective for older adults experiencing symptoms of depression and anxiety.  2) Participants who received Beating the Blues, in addition to treatment as usual (TAU), demonstrated significantly greater improvements in their depression and anxiety symptoms compared to those who received TAU alone, both at the end of treatment and during the follow-up period. |
| Gould et al., 2017 | Anxiety | No | Video only | CBT | 4 weeks | 20/20 | N/A | 1) Participants provided suggestions for improving video clarity and addressing individual differences, suggesting that while many found the BREATHE program beneficial, others indicated a preference for more personalized treatment approaches or face-to-face therapy. |
| Xiang et al., 2021 | Depression | No | Web-based | CBT | 8 sessions | Not reported | N/A | 1) Participants, including both homebound older adults and home care workers, reported that the internet-based cognitive behavioral therapy (iCBT) program, Beating the Blues (BTB), was effective in improving their knowledge and skills related to managing depression. They observed increased engagement in social activities and interactions, decreased negative thinking, and enhanced client-worker relationships.  2) Despite the positive impacts, participants faced significant challenges and technical difficulties with the iCBT program included poor usability of the program's web interface, accessibility issues (such as small font size and low volume), and the program content being perceived as non-engaging or irrelevant to older adults' experiences.  3) External assistance from home care workers or research assistants played a crucial role in facilitating the use of and engagement with the iCBT program. |
| Spek et al., 2007 | Depression | Yes | Web-based | CBT | 8 weeks, one session per week | 181/301 | N/A | 1) The study found that the internet-based psychotherapy intervention was effective in reducing symptoms of depression among older adults.  2) The study also identified challenges and barriers to the implementation of internet-based psychotherapy for older adults including issues related to technology use, such as difficulties with navigating online platforms and limited internet skills among some participants, suggesting the need for tailored support and training. |
| Staples et al., 2016 | Anxiety and Depression | Not reported | Web-based | CBT | 8 weeks | Research group 362/433; Clinic group 388/516 | N/A | Both the clinic group, which participated in the Wellbeing Plus Course through a public health setting, and the research group, participants from a randomized controlled trial, experienced significant symptom reductions in anxiety and depression at post-treatment, with results maintained at a 3-month follow-up. |
| Preschl et al., 2012 | Depression | Not reported | Offline platform | Life Review Therapy | 6 weeks | 36/40 | N/A | 1) Participants who received life-review therapy with computer supplements experienced significant decreases in depression and obsessive reminiscence, as well as improvements in well-being and self-esteem. |
| Chao et al., 2015 | General psychological distress | No | Game | Standing exercise | twice a week for 4 weeks | 30/32 | N/A | 1) Participants in the Wii Fit group exhibited significant improvements in balance and mobility, as well as a reduction in depression symptoms, after the 4-week intervention.  2) The study supports the idea that interventions designed around self-efficacy principles, such as setting achievable goals and providing positive feedback, can motivate older adults to participate in and benefit from physical activities like exergames. |
| Read et al., 2021 | Psychological distress with physical condition | Not reported | Web-based | CBT | 5 lessons in 8 weeks | 297/308 | N/A | 1) Participants experienced a significant reduction in depressive disorders in the group that received the iCBT program compared to the treatment as usual (TAU) group.  2) The iCBT program demonstrated high engagement and acceptability among participants, with high completion rates. Additionally, over 90% of participants felt the course was worth their time. |
| Villani et al., 2018 | Psychological distress with physical condition | Not reported | Web-based | Stress inoculation training | 10 sessions in 2 weeks | 29/40 | N/A | 1) Three months after the completion of the eHealth stress inoculation training (SIT) intervention, participants showed a significant reduction in emotional suppression and an increase in cancer-related emotional well-being.  2) Participants reported a good level of acceptance of the eHealth intervention. There was an observed increase in relaxation and a reduction in anxiety among the participants, further supporting the intervention's effectiveness in managing emotional experiences. |
| Fields et al., 2021 | General psychological distress | No | Robot | Shakespeare participatory art activity using the robot | 3 sessions | 13/15 | N/A | 1) Participants experienced significant reductions in depression, loneliness, and negative mood states.  2) The intervention's effects on depression varied between participants with and without dementia, indicating a potentially greater benefit for those without cognitive impairments. However, all participants, regardless of cognitive status, demonstrated overall improvements in well-being. |
| Benda et al., 2020 | Depression | Yes | Wearable technology | Not reported | One-week trial | 15/15 | N/A | 1) The intervention demonstrated high feasibility of use among older adults, with participants successfully completing 99% of their assigned tasks.  2) The study underscores the significance of completing usability assessments and involving target user groups in designing interventions that incorporate technology into care. This approach ensures the interventions are user-friendly and accessible to older adults, particularly those with mental health needs. |
| Bond et al., 2010 | Psychological distress with physical condition | No | Web-based | Problem solving therapy; Psychoeducation | 6 months | 62/62 | N/A | 1) Participants in the intervention group experienced significant improvements in depression, quality of life, social support, and self-efficacy compared to the control group.  2) The study supports the conclusion that web-based interventions can be an effective tool for improving the psychosocial health of older adults with diabetes. |
| Bennion et al., 2020 | General psychological distress | Not reported | Chatbot | Problem solving therapy | 1 session 20mins | 98/112 | N/A | 1) MYLO was more effective than ELIZA in reducing problem-related distress and received higher ratings for helpfulness and likelihood of reuse.  2) Both chatbots had below-average usability scores, but MYLO's usability was more strongly linked to user satisfaction and problem resolution.  3) Both chatbots reduced depression, anxiety, and stress scores, with improvements continuing over time, suggesting their potential for psychological support in older adults. |
| Shaunfield et al., 2014 | General psychological distress | No | VR | Not reported | 1 month | 15/21 | N/A | 1)Virtual trip led to a significant increase in the mental health of long-term care residents. There were promising trends showing decreased depression and improved social support among participants post-intervention, although these changes were not statistically significant.  2)This study demonstrated that virtual field trips are a feasible and engaging intervention for long-term care residents, offering a viable solution to enhance their quality of life and overcome barriers such as cost and environmental constraints. |
| O'moore et al., 2018 | Psychological distress with physical condition | Yes | Web-based | CBT | 10weeks | 37/44 | N/A | 1) The internet-based cognitive-behavioral therapy (iCBT) program significantly reduced depressive symptoms and psychological distress in older adults with knee osteoarthritis. Participants in the iCBT group also reported increased self-efficacy and improvements in osteoarthritis-related pain, stiffness, and physical function at the 3-month follow-up.  2) The iCBT program had high adherence rates (84%) and was well-received by participants, with most reporting satisfaction and a willingness to recommend the program to others. |
| Anguera et al., 2017 | Depression | Not reported | Game | Cognitive control interventions | 8 weeks of weekly sessions | 22/22 | N/A | 1) Both the Project EVO™ group and the Problem Solving Therapy (PST) group demonstrated significant improvements in mood and functional disability after treatment. The Project EVO group showed unique cognitive improvements, particularly in working memory and sustained attention, which were not observed in the PST group.  2) Participants using Project EVO experienced a notable and sustained decline in negativity bias (negative self-referential thinking), a benefit not evident in the PST group.  3) The EVO group achieved 100% compliance with the intervention, indicating the high acceptability and engagement of older adults with this therapeutic video game. |
| Yanez et al., 2015 | Psychological distress with physical condition | No | Web-based | Cognitive-behavioral stress management (CBSM) treatment | 10 weeks | CBSM group 30/35 ; Control group 31/36 | N/A | 1) Both the cognitive-behavioral stress management (CBSM) and health promotion (HP) interventions were well-received, with favorable weekly evaluations and exit surveys. Black participants gave higher ratings than non-Hispanic white participants, likely due to lower baseline access to supportive care resources.  2) The intervention was feasible, achieving over 85% retention at the 6-month follow-up and a 31.3% recruitment rate, consistent with challenges in enrolling advanced prostate cancer patients in clinical trials.  3) CBSM participants showed significant and clinically meaningful reductions in depressive symptoms compared to the HP group at the 6-month follow-up, with medium to large effect sizes. |
| Xiang et al., 2020 | Depression | No | Web-based | CBT and mindfulness | 8 sessions | 26/26 | N/A | 1) The iCBT program demonstrated feasibility, with a 55% session completion rate (average of 4.7 sessions out of 8).  2) Depressive symptoms significantly decreased from baseline to posttreatment, with a large effect size (Cohen’s d = 0.98). About 62.5% of participants achieved clinically meaningful reductions in symptoms.  3) Among those who completed the posttest assessment, 86% would recommend the program to others, and 71% found it an acceptable way to address depression. Participants who received external support reported higher satisfaction, with an average rating of 8.5 out of 10. |
| Ying et al., 2021 | General psychological distress | Yes | Web-based | CBT | 5 weeks | 111/127 | To make the lesson culturally appropriate to Chinese older adults, clinical psychologists collaborated with researchers in consultation with Chinese patients who have a history of depression and anxiety, with the majority of them being over 55 years old. | 1) Significant reductions were observed in depression, anxiety, general psychological distress, and functional disability post-treatment, with large effect sizes (Cohen's d ranging from 1.20 to 2.03). Improvements were sustained at the 1-month follow-up.  2) The program had a high completion rate of 87.4%, with participants completing an average of 4.74 out of 5 lessons. More than 85% of participants reported clinically meaningful improvements across all measures. |
| Rosenberg et al., 2010 | Depression | No | Game | Game and exercise | 12 week, three 35mins session a week | 19/22 | N/A | 1) The intervention was feasible, with an 86% retention rate over 12 weeks and an 84% adherence rate to the prescribed sessions.  2) Significant reductions in depressive symptoms were observed, with 37% of participants experiencing at least a 50% improvement. These improvements were sustained at the 20-24 week follow-up, where 53% reported similar reductions. |
| Nilsson et al., 2021 | Psychological distress with physical condition | No | Web-based | Not reported | 6 chapters | 93/120 | N/A | 1) The eHealth intervention with psychosocial support did not significantly reduce anxiety scores (HADS-A) compared to standard care in the full analysis.  2) No differences were found between groups in depressive symptoms (HADS-D) or mental health-related quality of life (SF-12 MCS), but physical quality of life (SF-12 PCS) declined more in the intervention group, likely due to higher rates of open repair surgery.  3) Only 50% of participants in the intervention group used the tool, with users being younger and more educated, suggesting barriers related to age and health literacy. |
| Read et al., 2020 | Psychological distress with physical condition | Yes | Web-based | CBT | 5 lessons in 8 week | 115/150(treatment group);152/152(control) | The intervention is designed for people aged over 60 year and has been shown efficacious for depression and anxiety. | 1) The iCBT group had significantly fewer cases of depressive disorders (5 cases) compared to the control group (15 cases) at the six-month follow-up, representing a 66% reduction in new cases.  2) Participants in the iCBT group experienced significant reductions in depressive symptoms immediately following the intervention.  3) The trial achieved a high retention rate of 96%, and 85% of participants reported being satisfied or very satisfied with the program. Over 94% said they would recommend it to others.  4) The iCBT intervention required minimal clinician contact (an average of 34 minutes per participant), demonstrating its potential as a scalable, cost-effective solution for preventing depression in older adults. |
| Šabanović et al., 2015 | Psychological distress with physical condition | Not reported | Robot | Not reported | Not reported | 5/5 | N/A | 1) Older adults with depression and therapists actively participated in the design process for socially assistive robots (SARs). While older adults preferred discussions and critiques over hands-on activities, they expressed strong interest in engaging with robot technologies.  2) Participants prioritized robots that were easy to use, low maintenance, and capable of personalized interactions, such as providing reminders, discussing daily topics, and offering encouragement. They emphasized the importance of robots providing social interaction, companionship, and emotional support, particularly during periods of loneliness or depression. |
| Easton et al., 2019 | Psychological distress with physical condition | No | Chatbot | Not reported | 2 co-design workshop | 5/6 | N/A | 1) Users preferred a multimodal interface (accessible via phone, tablet, TV, or laptop) and a human-like, customizable virtual agent. The ability to interact using both voice and text, along with integration with smart home devices and medical peripherals, was highlighted as desirable.  2) Participants found the prototype engaging and user-friendly. Many appreciated its potential as a companion providing emotional and practical support, particularly for individuals living alone or managing chronic conditions.  3) The virtual agent was seen as a valuable tool for promoting emotional well-being through motivational support and providing reassurance during health crises. Its ability to detect and respond to emotions like distress was considered crucial. |
| Henrique et al., 2021 | Depression | No | Mobile app | Psychoeducation and pleasurable activities | 17 weeks | 31/31 | The initiative (PROACTIVE) was developed to address the requirements of elderly individuals enrolled in the Family Health Strategy. | 1) The use of tablets enhanced session structure, stored participant data, and facilitated engagement through videos and interactive activities. Technology was well-received by both participants and health workers.  2) Older adults appreciated the structured protocol, which included multimedia content and behavioral activation activities. Most participants engaged actively, though some had reservations about the qualifications of non-specialist workers for severe depression cases.  3) Health workers, including nurse assistants and community health workers, followed the intervention protocol with high fidelity. Training, supervision, and the use of a tablet-based application were essential in ensuring adherence to the intervention’s structured approach. |
| Shah et al., 2018 | Depression | Not reported | Audio only; Offline platform | CBT | 4-week intervention | 37/51 | The intervention were developed using the client and therapist manuals employed in a cognitive-behavioral intervention research involving elderly individuals with depression. | 1) Both audio-based (ACBT) and computer-based (CCBT) cognitive behavioral therapies significantly reduced depressive symptoms compared to the delayed treatment (DT) control group. ACBT and CCBT groups showed greater decreases in interviewer-based depression scores (HRSD).  2) In the CCBT group, 53% experienced reliable reductions in depression scores, compared to 32% in the ACBT group and 13% in the control group.  Greater engagement with the intervention was associated with better outcomes, especially in the ACBT group.  3) Most participants in both ACBT (76%) and CCBT (88%) found the programs useful for learning techniques to improve mood, highlighting their acceptability. |
| Fortuna et al., 2018 | Psychological distress with physical condition | No | Mobile app | Not reported | 12 one-hour weekly module | 8/8 | N/A | 1) Older adults with serious mental illness (SMI) found the PeerTECH intervention highly acceptable. The combination of peer-delivered sessions and a smartphone app offered valuable support for managing both psychiatric and medical conditions.  2) The smartphone app complemented in-person sessions with features such as personalized daily tasks, medication reminders, peer-led videos, and secure text messaging. These tools helped reduce loneliness and promoted effective self-management beyond clinical settings.  3) PeerTECH proved to be a feasible and effective approach for integrating peer support with mobile health technology, especially for socially isolated or homebound older adults with SMI. |
| Morthland et al., 2020 | Depression | Not reported | Audio only; Offline platform | CBT | 4 weeks | Audio cognitive behavioral treatment: 66% completed the program; Computer cognitive behavioral treatment: 60% completed the program | Audio CBT (ACBT) and Computer CBT (CCBT), were developed specifically for older adults, incorporating user-friendly features like large fonts, simplified navigation, and examples relevant to older populations. | 1) Both ACBT and CCBT significantly reduced depressive symptoms. About 66% of ACBT participants and 60% of CCBT participants completed their respective programs, showing the feasibility of these approaches.  2) Participants in both groups reported high satisfaction, with 94% of ACBT users and 100% of CCBT users indicating they would recommend the program to others experiencing depression.  3) The interventions were designed to address barriers faced by older adults, such as limited internet access in rural areas and physical or sensory limitations. These low-cost, scalable approaches were well-received as potential solutions for underserved communities. |
| Knaevelsrud et al., 2017 | Post-traumatic stress disorder (PTSD) | Yes | Web-based | CBT | Two 45-minute writing assignments per week over a six-week | Treatment group: 37/47  Wait-list group: 44/47 | N/A | 1) The internet-based intervention, Integrative Testimonial Therapy (ITT), significantly reduced PTSD symptoms in older adults with childhood trauma compared to the waitlist control group.  2) Participants in the Integrative Testimonial Therapy (ITT) group experienced significant and sustained increases in quality of life and self-efficacy, showing moderate effect sizes.  3) The study highlighted the potential of ITT as a low-threshold, scalable intervention for older adults who face barriers to accessing traditional face-to-face therapy, such as mobility limitations or stigma. |
| Li et al., 2018 | Depression | No | Game | Not reported | One hour session per week for 6 consecutive weeks. | 102/102 | The difficulty levels of the exergames were set to a suitable level that meets the capacity of older adults. | 1) Exergames, compared to traditional exercise, had a stronger negative effect on subthreshold depression among older adults. The interactive and enjoyable features of exergames contributed to their effectiveness.  2) Positive emotions mediated the relationship between exercise type and reductions in depression. Exergames elicited higher levels of positive emotions, which further contributed to decreases in depressive symptoms.  3) Unlike positive emotions, self-efficacy did not significantly mediate the relationship between exercise type and depression. The short duration of the intervention (six weeks) and cultural factors may have influenced this outcome. |
| Chao et al., 2014 | General psychological distress | No | Game | Not reported | Twice per week for 8 weeks 60 minutes per session. | 7/7 | The intervention was adapted in accordance with the 'Exercising with Computers in Later Life (EXCELL) regimen' to more effectively cater to the requirements of elderly individuals. | 1) Depression symptoms and health-related quality of life (QOL) measures showed no significant differences after the intervention. Some participants reported frustration with the games, which may have affected these outcomes.  2) Although not statistically significant, there was a trend toward improved cognitive function after the intervention, particularly in orientation, recall, and calculation tasks.  3) Participants enjoyed the exergames, appreciating the music, visual elements, and group interactions. They expressed interest in continuing the program, highlighting its motivational and social benefits. |
| Fortuna et al., 2018 | Psychological distress with physical condition | No | Mobile app | Integrated Illness Management and Recovery (I-IMR) | Four times per month | 8/10 | The 'PeerTECH' initiative is derived from I-IMR, an evidence-based intervention designed for older adults. | 1) The PeerTECH intervention was found to be feasible and acceptable by both peer specialists and participants. Participants showed statistically significant improvements in psychiatric self-management skills, as measured by the Illness Management and Recovery Scale (IMRS).  2) There were also improvements in medical self-management skills, self-efficacy for managing chronic health conditions, hope, quality of life, and empowerment, although not all were statistically significant.  3) The smartphone app reinforced skills learned during in-person sessions and provided personalized support, medication reminders, and HIPAA-compliant chat features. |
| Silfvernagel et al., 2018 | Anxiety and Depression | Not reported | Web-based | CBT | 8 weeks | Treatment group: 11/33 | The treatment was designed to tailor individuals' needs and symptoms, aiming to equip them with the information and skills they require. | 1) Individually tailored internet-based cognitive behavior therapy (ICBT) significantly reduced symptoms of anxiety and depression in older adults compared to a control group receiving general weekly email support.  2) Nearly half (45.5%) of participants in the treatment group were classified as responders, showing clinically significant improvements, while only 3% experienced symptom deterioration.  3) Cognitive flexibility, as measured by the Wisconsin Card Sorting Test, was a significant predictor of treatment outcomes. Better cognitive flexibility was associated with greater reductions in symptoms. |
| Dear et al., 2013 | Depression | Yes | Web-based | CBT | 8 weeks with 5 lessons in total | 16/20 | The treatment protocol for depression and anxiety was modified to incorporate age-appropriate case examples and skill demonstrations tailored specifically for older adults. | 1) At post-treatment, 58% of participants achieved remission for depression (PHQ-9 scores < 10), and 58% showed a 50% reduction in symptoms. These outcomes were maintained at follow-up.  2) 82% of participants found the program worthwhile and would recommend it to others, reflecting high levels of acceptability.  3) The intervention required an average of 73.75 minutes of clinician support per participant, highlighting its cost-effectiveness and scalability. |
| Wada et al., 2006 | General psychological distress | No | Robot | Not reported | Two days per week for about one hour at a time | 10/14 | N/A | 1) Interaction with the therapeutic seal robot "Paro" significantly improved the mood of elderly participants, as measured by face scale evaluations. It also encouraged communication among residents and caregivers, fostering a more positive social atmosphere.  2) Participants willingly engaged with Paro, showing affection and treating it like a companion. They gave it names and discussed it with others, making it a common topic for conversation and connection.  3) Observations and preliminary physiological tests indicated that interacting with Paro reduced stress among participants, including those with dementia. |
| Schneider et al., 2003 | Psychological distress with physical condition | No | VR | Not reported | Not reported | 16/16 | N/A | 1) The virtual reality (VR) intervention significantly reduced state anxiety levels immediately following chemotherapy sessions (p = 0.10), demonstrating its effectiveness as a distraction technique.  2) All participants found the VR intervention easy to use, experienced no cybersickness, and 100% indicated they would use it again. Participants preferred chemotherapy sessions with VR compared to without it.  3) Participants used the VR device throughout the chemotherapy sessions, averaging 78 minutes of use, despite perceiving the duration as shorter, which suggests high engagement. |
| Jones et al., 2016 | Anxiety | Yes | Web-based | CBT | 7 modules | Treatment group: 22/24 Controlled  WLC group: 19/22 | N/A | 1) Participants in the internet-delivered cognitive behavioral therapy (ICBT) group showed significant reductions in generalized anxiety (GAD-7) and depression (PHQ-9) scores compared to the waitlist control group.  2) Higher pre-treatment credibility ratings for the ICBT program predicted faster reductions in anxiety symptoms over time, while treatment expectancy was not a significant predictor. |
| Muroi et al., 2020 | Anxiety | Not reported | Game | Not reported | Not reported | 42/42 | The puzzle game was designed with features suited to older adults, such as slow-paced gameplay, low stimulation, minimal judgment of winning or losing, and nostalgic or calming visual and auditory elements. | 1) The gamified puzzle intervention significantly reduced immediate (state) anxiety levels (S-AI) among elderly participants in the experimental group compared to the control group. Long-term (trait) anxiety levels (T-AI) were reduced but not as significantly.  2) Alongside self-reported anxiety reductions, skin conductivity (SC/GSR) measurements indicated decreased physiological arousal, supporting the effectiveness of the intervention. |
| Zou et al., 2012 | Anxiety | Yes | Web-based | CBT | 5 sessions | 22/22 | The internet-delivered cognitive-behavioral therapy content underwent changes to incorporate age-appropriate scenarios and skill-related illustrations. | 1) Participants saw a sharp decline in anxiety symptoms, with improvements lasting three months post-treatment. Stress and depression also improved, though some depressive symptoms returned at follow-up.  2) 100% of participants completed all lessons, and 95% followed up, showing strong participation and retention.  3) Therapy required just 78 minutes of clinician time per participant, making it accessible and scalable. |
| Chen et al., 2020 | Depression | Yes | Web-based | CBT | Not reported | 45/47 | N/A | 1) Many participants found the platform overwhelming due to navigation issues and usability challenges. These technological frustrations exacerbated existing insecurities and made them feel even more isolated or incapable.  2) Despite the challenges, many participants found the social interaction in the program beneficial. Engaging with peers fostered a sense of connection, reduced loneliness, and reinforced positive behavior change through shared experiences.  3) The study highlights the need for age-friendly digital health interventions that prioritize usability, clear guidance, and ongoing support. Simplifying the interface, pacing the program according to participants’ needs, and ensuring accessible training materials could improve effectiveness and reduce frustration. |
| Hwang et al., 2021 | Depression | No | Web-based | Not reported | Not reported | 159/197 | N/A | 1) Older adults who read messages on an online support forum experienced significant reduction in depressive symptoms over a year, even more than those with strong offline social networks.  2) Unlike message consumption, writing messages did not significantly reduce depressive symptoms, suggesting that passive engagement (reading) may be more beneficial than active participation.  3) The effect of reading online messages was greater than offline social connectedness in reducing depression, emphasizing the unique role of digital support networks for older adults. |
| Orr et al., 2020 | Anxiety and Depression | Yes | Mobile app | Cognitive restructuring | 8 weeks | 3/3 | N/A | 1) Older adults actively used the IntelliCare mobile intervention, showing frequent app engagement and strong interaction with coaches.  2) Participants significantly improved in depressive and anxiety symptoms, with some progressing to full remission by the end of the intervention.  3) Different participants benefited in unique ways, from improved mood and self-esteem to better sleep and social engagement, showing the flexibility of mobile interventions.  4) Human support through text-based coaching helped participants navigate challenges, stay engaged, and apply skills to real-life situations. |
| Dear et al., 2015 | Anxiety and Depression | Not reported | Web-based | CBT | 5 lessons in 8 weeks | 47/54 | The online courses, including "Managing Stress and Anxiety" and "Managing Your Mood” offered case stories and skill use examples tailored to the appropriate age group. | 1) Participants showed significant reductions in anxiety and depression, with improvements maintained at a 3-month follow-up.  2) Over 90% participants found the course worthwhile, and most participants completed at least three out of five lessons.  3) The intervention led to improved health-related quality of life, though costs were slightly higher during treatment. |
| Li et al., 2016 | Depression | Not reported | Game | Not reported | 1 hour per session, 1session per week 6 consecutive weeks | 49/59 | Participants will have access to one of the two exergames from Wii Sports, specifically Wii Bowling and Wii Golf, both of which were used in the high playfulness (HP) condition. To prioritize safety and address concerns related to energy expenditure and age, the game difficulties were calibrated to match the capabilities of older adults. | 1) Both high-playfulness (Wii Sports) and low-playfulness (Wii Fit) exergames significantly reduced subthreshold depression in older adults. Participants in the high-playfulness group experienced greater increases in positive emotions, but playfulness did not significantly impact depression or self-efficacy.  2) Both groups saw significant gains in self-efficacy, though these improvements did not differ between the two conditions.  3) While exergames improved mood immediately, longer intervention periods may be needed to see lasting effects on depression. |
| Similä et al., 2018 | General psychological distress | No | Mobile app | Web‐based acceptance and commitment therapy (ACT) | 46 text and audio exercises and an introductory video | 4/7 | N/A | 1) While some older adults found the mobile mental wellness app useful and engaging, many faced barriers in adoption due to low digital literacy and usability challenges. Many older users struggled with smartphone navigation, small screen sizes, and technical issues, limiting sustained engagement.  2) Participants who engaged with the app reported improved sleep, reduced stress, and better mood regulation, suggesting benefits for mental wellness. The app was more effective when integrated into group activities rather than used independently, as group support helped with motivation and learning.  3) Simplifying interface design, providing structured guidance, and offering alternative formats (e.g., CDs or tablets) could improve accessibility and usability for older adults. |
| Tomasino et al., 2017 | Depression | Not reported | Web-based | CBT | 8-week online intervention | 40/47 | In this study, we employed MoodTech, an 8-week online intervention for depression rooted in Cognitive Behavioral Therapy (CBT) principles, tailored for individuals aged 65 and older. | 1) Both individual (III) and peer-supported (II+PS) internet interventions significantly reduced depressive symptoms compared to a waitlist control.  2) While depression improved, the intervention did not significantly reduce social isolation, though some participants reported increased social support.  3) Participants engaged well with the platform, but usability scores were below ideal, particularly in the peer-supported group. |
| Chiu & Wu, 2019 | General psychological distress | Not reported | Web-based | ICT-communication and ICT-entertainment | Once a week for 12 weeks | 54/57 | Technology devices, like touchscreen tablets equipped with tailored assistance tools such as tablet holders and sensor pens designed specifically for particular disabilities, were employed. | 1) Older adults using entertainment apps (YouTube) showed greater improvements in mental and physical health, social support, and happiness compared to those using communication apps or receiving usual care.  2) Both ICT-communication and ICT-entertainment groups significantly decreased depressive symptoms, but the entertainment group saw the largest reduction.  3) ICT-entertainment users reported stronger social support from health care workers and higher overall satisfaction with their support network.  4) Using communication apps (e.g., Line) was more difficult for older adults, especially those with physical limitations, reducing its effectiveness compared to entertainment-based ICT. |
| Wahbeh, 2018 | Depression | No | Web-based | Internet Mindfulness Meditation Intervention (IMMI) | 6 one-hour weekly sessions with 20 min of home practice meditation between sessions | 36/50 | N/A | 1) IMMI participants showed strong improvements in depressive symptoms compared to the waitlist control, with effects sustained for seven weeks post-intervention.  2) Participants also experienced reduced perceived stress, improved sleep, and decreased pain severity, enhancing overall well-being.  3) While participants rated IMMI positively, adherence to the full program was low, likely due to external stressors (e.g., wildfires affecting participants' lives).  4) The intervention was effective with limited staff involvement, making it a low-cost, scalable mental health tool for older adults. |
| Spek et al., 2008 | Depression | Yes | Web-based | CBT | 10 weekly group sessions | Internet group:58/102  group course group: 66/99 waiting list group: 66/100 | N/A | 1) Internet-based CBT remained significantly more effective than a waitlist control even one year after treatment. 62% of internet CBT participants scored below the depression threshold at follow-up, compared to 45% in group CBT and 38% in the control group.  2) 37% of participants did not complete follow-up measures, and the study sample was more educated than the general population, indicating barriers to widespread adoption.  3) Internet-based CBT offers a low-cost, scalable intervention that can reach more older adults and help prevent full-blown depression in those with subthreshold symptoms. |
| Proyer et al., 2014 | Depression | Not reported | Web-based | Positive psychology interventions | Not reported | 163/510 | N/A | 1) Three interventions (gratitude visit, three good things, and using signature strengths) significantly boosted happiness, with effects lasting up to six months.  2) Three funny things and using signature strengths interventions led to significant reductions in depressive symptoms, though effects varied by time point.  3) The findings suggest positive psychology interventions work similarly for older adults as they do for younger populations, supporting their use in digital formats.  4) Self-guided online interventions could serve as accessible, low-cost tools to enhance well-being and reduce depression in older adults. |
| Gamito et al., 2010 | Post-traumatic stress disorder (PTSD) | No | VR | Not reported | 12 sessions | 9/10 | N/A | 1) Veterans in the VRET group showed an 8% reduction in PTSD symptoms and a 40% decrease in depression, outperforming the imagination exposure (EI) and waitlist groups.  2) VRET participants reported less anxiety and somatization, with significant improvements in mental health after 12 sessions.  3) Compared o Traditional Exposure Therapy, VRET showed at least similar benefits to imagination-based exposure therapy (EI), suggesting VR can be an effective alternative for PTSD treatment. |
| Lappalainen et al., 2022 | General psychological distress | Not reported | Web-based | Web-Based Acceptance and Commitment Therapy | 12 sessions | 106/149 | N/A | 1) The CareACT intervention significantly reduced depressive symptoms compared to institutional rehabilitation and caregiver associations at four months, but effects were not fully sustained at 10 months.  2) CareACT participants showed greater reductions in thought suppression, a dysfunctional coping strategy linked to depression and anxiety, compared to other groups.  3) 83% of participants would recommend CareACT, and participants completed an average of 87% of the program, showing strong engagement. |
| Titov et al., 2016 | Anxiety and Depression | Not reported | Web-based | Not reported | 5 lessons | 433/459 | N/A | 1) All groups (clinician-guided, self-guided with interview, and fully self-guided) showed large reductions in anxiety and depression which were sustained at a 3-month follow-up.  2) Clinician guidance did not lead to better outcomes; self-guided participants achieved similar symptom reductions and satisfaction levels.  3) Across all groups, most participants completed the full intervention, with 95%+ reporting satisfaction and saying they would recommend the program.  4) The self-guided groups needed little to no clinician contact, making this a cost-effective and scalable mental health intervention. |
| Gould et al., 2019 | Anxiety | No | Video only | CBT | 4 weeks of video lessons plus phone calls; continue practicing relaxation on their own for week 5−8 | BREATHE group: 13/20 WLC: 20/20 | N/A | 1) The BREATHE program led to a significant reduction in anxiety symptoms, particularly cognitive anxiety (e.g., worry), compared to the waitlist control.  2) Participants also experienced reduced depressive and somatic symptoms, though activity engagement did not significantly improve.  3) 35% of participants dropped out, citing external commitments or dissatisfaction with the intervention format (e.g., expecting higher-tech content).  4) The intervention was effective with only brief telephone coaching, making it a low-cost, scalable option for treating anxiety in older adults. |
| Titov et al., 2015 | Depression | Not reported | Web-based | CBT | 5 sessions in 8 weeks | Treatment group: 23/27  WCL: 22/25 | N/A | 1) Participants receiving therapist-guided iCBT showed large reductions in depression and anxiety with improvements maintained at 3-month and 12-month follow-ups.  2) 70% completed the full 8-week program, and most participants rated the intervention as acceptable and effective.  3) While iCBT had slightly higher costs than usual care ($52 more per participant), it was considered cost-effective based on Quality-Adjusted Life Years (QALYs) and willingness-to-pay thresholds.  4) Participants received brief weekly therapist support via phone or email, with an average of 45 minutes per participant, making it a low-cost, scalable option. |
| Xiang et al., 2024 | Depression | No | Web-based | CBT | 9 sessions in 10 weeks | 64/70 | The program content is tailored to the needs of older adults, and coaching support is provided to ensure accessibility and engagement. | 1) The Empower@Home program significantly reduced depressive symptoms in older adults  2) Most participants completed all sessions, and the program received high usability and acceptability ratings. Coaching provided by trained laypersons effectively supported participant engagement and program adherence.  3) Participants in the treatment group also experienced notable improvements in anxiety, anger, social isolation, insomnia, and pain, although between-group differences in these secondary outcomes were not statistically significant. |
| Kong et al., 2024 | Depression | Yes | Web-based | CBT | 5 lessons in 5 weeks | ICBT Group: 83/105; CBT Group: 65/104 | N/A | 1) ICBT significantly reduced depressive symptoms among older adults with subthreshold depression (sD) in long-term care (LTC) settings, with improvements maintained at 6- and 12-month follow-ups.  2) ICBT outperformed both group CBT and the waitlist control in reducing PHQ-9 and GAD-7 scores at postintervention and follow-up, indicating stronger effects on depression and anxiety. |
| Danieli et al., 2022 | Anxiety | Not reported | Chatbot | CBT | 8 weeks | 45/60 | N/A | 1) Significant within-group improvements were observed in group receiving (traditional therapy combined with AI agent, including reduced stress (PSS), psychological distress (SCL-90-R), and improved coping and physical health (OSI).  2) Satisfaction and perceived usefulness were highest in Group 2, suggesting that participants valued the combination of human therapy and AI support more than either alone.  3) No significant differences were found between groups at post-treatment or follow-up, meaning adding the AI agent did not statistically outperform traditional therapy or the control when comparing across groups. |
| Chou et al., 2024 | Anxiety and Depression | Yes | Chatbot | Psychoeducation | 4 weeks | 35/35 | The chatbot was built on LINE, the most widely used messaging platform among older adults in Taiwan, and was designed with button-based interactions to enhance user-friendliness. Educational videos in the program were specially created by a rehabilitator, featuring age-appropriate movements tailored to older adults. | 1) Participants aged 65 and older showed a statistically significant improvement in loneliness, an effect that was not observed in the younger group (aged 55–65). While loneliness improved among older adults, there were no statistically significant changes in depression or anxiety in either age group.  2) Older adults rated the chatbot highly in terms of usability and satisfaction, reflecting the success of its age-friendly design. |
| Nordgren et al., 2024 | Depression | Yes | Web-based | CBT | 6–10 modules in 10 weeks | 93/101 | The treatment modules were modified to align with older adults’ preferences. Both the content and language were adapted to their context—language was simplified and made more legible, and age-appropriate examples were incorporated. | 1) The treatment group showed greater improvements in depression compared to the control group, as measured by the Geriatric Depression Scale (GDS-15) and the Beck Depression Inventory-II (BDI-II), indicating that tailored ICBT significantly reduced depressive symptoms.  2) The intervention demonstrated strong feasibility and engagement among older adults, with a low dropout rate of only 8% at post-treatment and an average completion of 76% of prescribed modules. |
| Primavera et al., 2024 | Depression | Not reported | VR | Not reported | 2 sessions/week in 3 months | 21/21 | N/A | 1) The study reported a 100% completion rate among older adults (ages 58–75) participating in the VR-based cognitive remediation (VR-CR).  2) While there were some improvements in both cognitive test scores and depressive symptoms (PHQ-9) in the experimental group, none reached statistical significance compared to the control group. |
| Lappalainen et al., 2022 | General psychological distress | Not reported | Web-based | ACT | 12 weeks | 51/59 | N/A | 1) The CareACT group showed significantly greater reductions in depressive symptoms than both the institutional rehabilitation and caregiver association groups at the 4-month follow-up, with a medium effect size (d = 0.70). A the 10-month follow-up, the benefits in the CareACT group were maintained but with smaller effect sizes (d = 0.32 vs rehab; d = 0.36 vs association), and the differences were no longer statistically significant.  2) The CareACT group had an 86.4% completion rate, and participants completed on average 87.4% of the program. 83% would recommend it to others, and nearly 70% felt it helped them cope better. |
| Xiang et al., 2024 | Depression | No | Web-based | CBT | 9 sessions in 10 weeks | 145/148 | Empower@Home is an older adult–centered iCBT program specifically designed for ease of use, featuring custom elements such as large buttons, clear visuals, and minimal text. | 1) Participants experienced a significant decline in depressive symptoms.  2) Participants consistently praised the age-friendly design interface that addressed common usability challenges for older adults.  3) Nearly all participants (145 out of 148) completed the full nine-session iCBT program, indicating strong feasibility and engagement among older adults. |
| Fan et al., 2022 | Depression | No | VR | Not reported | 8 weeks | 62/62 | N/A | 1) The intervention significantly improved self-esteem and perceived mastery among older adults in the experimental group compared to the control group. However, there were no significant improvements in depression and isolation compared to the control group.  2) All participants in the experimental group completed the 8-week program, demonstrating high feasibility and acceptability of the combined 3D VR and hands-on horticultural activities for community-dwelling older adults. |
| Borghouts et al., 2022 | Anxiety and Depression | No | Web-based | CBT | 3 months | 23/30 | N/A | 1) Participants valued three main types of support: technical help, guided reminders to use the mental health app (*myStrength*), and social support to feel connected during the program.  2) After the program, the percentage of participants who felt confident using technology rose from 31% to 73%, and 79% reported being satisfied with the digital literacy training. |
| Xiang et al., 2023 | Depression | No | Web-based | CBT | 9 sessions in 10 weeks | 3/4 | The platform was designed with age-friendly features such as large buttons, icons with text descriptions, high-contrast color schemes, and intuitive navigation. In addition, the program includes a large-print user workbook, recognizing that older adults are more likely to be familiar with and comfortable using print materials. | 1) Participants showed a downward trend in depression scores (PHQ-9) over the course of the program, indicating potential effectiveness for reducing depressive symptoms, though the sample was too small for inferential statistics.  2) In a comparative usability evaluation with 10 older adults, the Empower@Home program received significantly higher usability scores (mean SUS = 78) than two established iCBT programs (Beating the Blues and MoodGym. In a 10-week pilot test with four low-income, homebound older adults, all participants completed at least 8 of the 9 sessions (three completed all 9), suggesting excellent engagement and feasibility in a high-need population. |
| Xiang et al., 2024 | Depression | No | Web-based | CBT | 9 sessions in 10 weeks | 94/103 | Empower@Home is a digital mental health intervention (DMHI) specifically tailored for older adults. Participants received large-print workbooks designed for ease of use, which included session summaries and guided exercises to support their engagement with the program. | 1) Participants reported a significant decrease in depression scores (PHQ-9) from pretest to posttest and follow-up, along with significant reductions in anxiety and improvements in social support and loneliness.  2) The program demonstrated excellent feasibility and adherence, with 91.3% of participants (94 out of 103) completing all nine sessions and a 93% retention rate at posttest. |
| Giordano et al., 2024 | Depression | No | Mobile app | Not reported | 12 weeks | 14/20 | The app was designed to support biographic-narrative work between residents of long-term care homes and older volunteers through virtual excursions, with specific adaptations to accommodate the communicative and cognitive abilities of older adults. | 1) Residents in the intervention group showed a significant increase in social participation after the intervention, which remained stable at the 3-month follow-up.  2) Volunteers experienced a significant improvement in mental quality of life and participation immediately after the intervention.  3) There were no significant changes in depressive symptoms among residents, and the study could not confirm the intervention’s preventive effect on depression.  4) The study demonstrated that trained volunteers could successfully deliver a tablet-based biography intervention, and older adults were able to engage with the digital format with support. |
| Gould et al., 2024 | Anxiety | No | Video only | CBT | 4 weeks | BREATHE Group: 22/27; Healthy Living Group: 24/29 | N/A | 1) The two interventions (BREATHE and Healthy Living) did not differ significantly in their effects on overall anxiety symptoms or functioning.  2) The psychoeducation group (Healthy Living) showed a significantly greater reduction in somatic anxiety symptoms compared to the BREATHE group.  3) Many BREATHE participants reported using breathing and relaxation techniques in real-world situations (e.g., during stress, medical visits), and personalizing the practice routines helped sustain use over time. |
| Park & Kim, 2022 | Depression | No | Voice assistant | Not reported | 2 months | 291/317 | N/A | 1) Depression scores significantly decreased after two months of AI speaker use across all participants. Both frequent and intermittent users showed improvement, but the change was particularly notable in frequent users.  2) Loneliness significantly decreased only among frequent users (those who used the speaker 5+ times per week). Intermittent users showed no significant change in loneliness.  3) Older adults who were more lonely at baseline tended to use the AI speaker more frequently, suggesting they may have viewed it as a source of social connection.  4) While depression decreased in both groups, only loneliness showed a clear difference between frequent and intermittent users, indicating that consistent use may be key to reducing social isolation. |
